# Digital outdoor exercise program for obese patients with type 2 diabetes mellitus: a non-inferiority randomized controlled trial

**DOI:** 10.3389/fendo.2025.1654129

**Published:** 2025-07-31

**Authors:** Jian Cui, Qiang Liu, Lihua Huang, Haoyan Yu

**Affiliations:** ^1^ Department of Physical Education, China University of Geosciences (Beijing), Beijing, China; ^2^ Department of Rehabilitation, Huashan Hospital Affiliated to Fudan University, Shanghai, China; ^3^ Department of Rehabilitation, Shanghai Sixth People’s Hospital Affiliated to Shanghai Jiao Tong University School of Medicine, Shanghai, China; ^4^ Department of Radiology, Shanghai Sixth People’s Hospital Affiliated to Shanghai Jiao Tong University School of Medicine, Shanghai, China

**Keywords:** type 2 diabetes mellitus, obesity, digital exercise program, outdoor exercise, randomized controlled trial

## Abstract

**Background:**

Obesity and physical inactivity exacerbate type 2 diabetes mellitus (T2DM), whereas regular exercise improves glycemic control, fitness, and quality of life. However, many patients face barriers to attending clinic-based exercise programs. Digital health interventions could increase access and adherence by enabling guided outdoor exercise via smartphone. It remains unclear if a digital program can achieve outcomes comparable to traditional supervised exercise in obese adults with T2DM.

**Objective:**

We aimed to evaluate the noninferiority of a 12-week digital outdoor exercise program, delivered via mobile app, compared to a standard clinic-based exercise intervention in obese adults with T2DM. The primary outcome was the change in glycated hemoglobin (HbA1c). Secondary outcomes included changes in body mass index (BMI), physical fitness, and quality of life. Adherence and cost-effectiveness were also assessed.

**Methods:**

We conducted a randomized controlled noninferiority trial at a single tertiary center. A total of 240 obese adults with T2DM were randomly assigned to either the digital outdoor exercise program (DOE) or a clinic-based exercise program (CBE). The digital intervention provided personalized aerobic and resistance exercise routines via a smartphone app with remote coaching, while the clinic group attended on-site supervised exercise sessions of similar frequency and intensity. Outcomes were measured at baseline and 12 weeks. The noninferiority margin for HbA1c was set at 0.4%. Analyses were performed on an intention-to-treat basis.

**Results:**

A total of 240 obese adults with T2DM were randomized equally into DOE and CBE groups. After 24 weeks, both groups achieved significant, comparable reductions in HbA1c (DOE: -1.56 ± 0.17%, CBE: -1.50 ± 0.17%), BMI, waist circumference, and improved physical fitness, with no significant between-group differences. The DOE intervention demonstrated significantly lower costs (14,787.30 CNY) compared to CBE (17,920.05 CNY; p<0.001). Adherence was high in both groups, with similarly low adverse event rates.

**Conclusions:**

The 12-week smartphone-based outdoor exercise program was noninferior to a clinic-based program in improving HbA1c and BMI, and it produced similar gains in fitness and quality of life in obese adults with T2DM. Higher adherence in the digital intervention and its lower delivery cost indicate that digital exercise programs can be a cost-effective, scalable alternative to clinic-based interventions for managing T2DM.

**Clinical trial registration:**

https://www.chictr.org.cn/, identifier ChiCTR2500104389.

## Background

Type 2 diabetes mellitus (T2DM) and obesity are global health challenges, with China currently having the largest diabetic population globally, accounting for approximately 25% of cases (1). The prevalence of diabetes among Chinese adults rose dramatically from less than 1% in 1980 to over 11% by recent estimates (1, 2), posing significant economic burdens and strain on healthcare systems (1, 3). Effective management of T2DM requires lifestyle interventions, particularly structured exercise programs, known to significantly reduce glycated hemoglobin (HbA1c) levels, thus lowering the risk of diabetes-related complications (4). However, traditional clinic-based exercise rehabilitation, though effective, often demands substantial healthcare resources, specialized personnel, and physical facilities, making it less accessible due to geographic, economic, and logistical constraints, particularly evident within China’s extensive and varied landscape (3, 5).

Digital health innovations offer a promising alternative by utilizing telemedicine and mobile health technologies to deliver remote, structured exercise programs (tele-exercise), potentially overcoming these barriers. Tele-exercise interventions enable patients to undertake guided exercises at home or in outdoor community settings, supported remotely via smartphones, wearable technologies, and video conferencing. This delivery model provides flexibility, improves adherence, and can engage patients continuously, bridging intervals between clinical visits (5, 6). Importantly, such remote programs have demonstrated efficacy comparable to traditional, clinic-based programs in terms of improving glycemic control, lipid profiles, body composition, physical function, and quality of life (6, 7). For instance, internet-based exercise interventions have shown significant reductions in HbA1c levels, improvements in aerobic capacity, and overall diabetes management, matching the outcomes observed with supervised, facility-based programs (8, 9).

Economically, digital exercise interventions present additional advantages by substantially reducing the need for facility utilization and direct personnel supervision, thereby lowering costs associated with program delivery (10). Economic analyses of physical activity programs in type 2 diabetes management consistently report cost-effectiveness, with some telemedicine-based interventions achieving cost-savings while maintaining clinical effectiveness (10, 11). In contexts like China, where diabetes prevalence is high and specialist healthcare resources are unevenly distributed, scalable and cost-effective digital health solutions are especially advantageous, promising improved access and sustainability (3, 5).

Given existing evidence supporting the efficacy and economic benefits of digitally delivered exercise programs (6, 7, 10), employing a non-inferiority trial design is justified. This approach seeks to establish whether a digitally delivered exercise intervention provides outcomes not clinically worse than traditional clinic-based rehabilitation. Demonstrating non-inferiority could significantly support broader integration of tele-exercise models into diabetes management frameworks, particularly within healthcare systems challenged by resource constraints, such as China’s. The purpose of this study is to evaluate the clinical outcomes and cost-effectiveness of this digital outdoor exercise program, in comparison with traditional clinical-based exercise for obese patients with T2DM in China.

## Methods

### Study design and setting

This study was designed as a single-center, parallel-group, assessor-blinded, non-inferiority randomized controlled trial conducted at a tertiary care hospital in Shanghai, China. Patients were recruited from endocrinology outpatient clinics at the facility. Each participant was involved for approximately 24 weeks, comprising a 12-week active intervention and a subsequent 12-week follow-up period. Study assessments occurred at baseline, mid-intervention (6 weeks), at the completion of intervention (12 weeks, primary endpoint), and at the end of follow-up (24 weeks). Ethical approval for the study was obtained from the hospital’s institutional ethics committee (2022-KY-041), and the trial was registered in the Chinese Clinical Trial Registry (ChiCTR2500104389), in accordance with the latest CONSORT guidelines for clinical trial reporting (12).

### Participants

#### Inclusion criteria

Participants were eligible if they were aged between 18 and 70 years, had a confirmed diagnosis of type 2 diabetes mellitus lasting at least one year, and met the obesity criterion defined by Chinese standards (BMI ≥28 kg/m²) (2). Additional inclusion criteria included baseline HbA1c levels ranging between approximately 7% and 10%, a sedentary or minimally active lifestyle (less than 150 minutes of moderate-intensity physical activity per week over the past three months), medical clearance to participate in moderate-intensity exercise, and having access to and basic proficiency with a smartphone or tablet device.

#### Exclusion criteria

Exclusion criteria comprised having type 1 diabetes or secondary diabetes; severe diabetic complications precluding safe exercise participation, such as proliferative retinopathy, significant peripheral neuropathy with fall risks, or active diabetic foot ulcers; unstable or severe cardiovascular diseases such as recent myocardial infarction (within six months), uncontrolled hypertension (blood pressure exceeding 180/100 mmHg), or advanced heart failure (NYHA class III-IV); significant musculoskeletal or joint disorders impeding exercise; chronic kidney disease stages 4–5; pregnancy or intentions of becoming pregnant during the study; current participation in another structured diabetes or exercise program; and inability or unwillingness to adhere to the intervention protocol.

### Interventions

All participants underwent thorough pre-participation screening by a physician to ensure they could safely engage in the exercise program. This screening evaluated cardiovascular health, musculoskeletal conditions, and overall fitness for aerobic exercise. We designed the aerobic exercise regimen to be progressive but adaptable to individual capacity. For older participants, the starting intensity was set at a modest level and increased more gradually, with close monitoring of their response. Throughout the program, we continuously monitored participants’ heart rate and perceived exertion. Study staff checked in regularly (by phone or in-person visits) to ensure older individuals were tolerating the exercise well. If any participant, particularly an elderly one, showed signs of over-exertion or discomfort, the protocol was adjusted: for example, allowing extra rest, reducing the speed/incline of walking sessions, or extending the time at a lower intensity before progressing. We also included mandatory warm-up and cool-down periods in every session to reduce the risk of strain or injury.

#### Digital outdoor exercise program

Participants assigned to this arm received a structured 12-week exercise intervention facilitated by a smartphone application. The exercise program integrated aerobic and resistance training aligned with established diabetes exercise guidelines. Participants were instructed to achieve a weekly total of approximately 150 minutes of moderate-intensity aerobic exercises outdoors, complemented by two sessions per week of resistance exercises targeting major muscle groups. Participants received instructional videos, exercise planning, adherence monitoring, motivational reminders, and safety alerts through the app. Exercise intensity was monitored via the app-integrated wearable devices or phone accelerometers, and remote coaching was provided weekly through video or phone calls. Coaches adjusted exercise prescriptions based on individual performance and feedback. Full details of the digital intervention are provided in **Supplementary Materials**.

In the DOE group, each participant was provided with the same model of wearable heart rate sensor to use during exercise sessions. Specifically, we supplied a chest-strap heart rate monitor that paired with our exercise app.

#### Clinic-based exercise program

The control arm consisted of traditional clinic-based rehabilitation at the hospital’s rehabilitation facility, involving supervised sessions by physiotherapists or exercise specialists three times weekly, each lasting about 60 minutes. These sessions comprised combined aerobic exercises (e.g., treadmill, stationary cycling) and resistance training (e.g., gym equipment, free weights), conducted at moderate intensity monitored through heart rate measurements. Participants were also encouraged to perform two additional home-based aerobic exercises weekly. Compliance was monitored through exercise diaries reviewed by therapists. Detailed descriptions are provided in **Supplementary Materials**.

### Randomization, allocation, and blinding

After completing all baseline assessments and consent procedures, participants will be randomly allocated to either the digital program or the clinic-based program. Randomization will be performed in a 1:1 ratio using a computer-generated sequence with random block sizes of 4–6 to ensure approximately equal numbers in each group over time. The random sequence will be generated by an independent biostatistician or research coordinator who is not involved in participant enrollment or intervention delivery. To further assure allocation concealment, the assignments will be prepared using sequentially numbered, opaque sealed envelopes containing the group allocation. When a participant is eligible and ready to enroll, the study coordinator will open the next envelope to determine the group allocation. This process takes place only after baseline data collection to prevent any potential bias or predictions about assignment. Blinding in this trial is partial due to the nature of the interventions. Participants and intervention facilitators cannot be fully blinded to group assignment: those in the digital arm will know they are using an app-based program, and those in the clinic arm will be attending in-person sessions, so they are inherently aware of their intervention. Similarly, the exercise physiotherapists providing the interventions must know which program they are administering. However, outcome assessment and data analysis will be conducted in a blinded manner to minimize bias. Research staff members who conduct follow-up measurements will not be involved in the intervention delivery and will remain blinded to each participant’s group assignment. Participants will be instructed not to reveal any details of their program to the assessors during these evaluations. Key outcome measures, notably laboratory analyses like HbA1c, will be performed in a blinded fashion in the hospital’s central lab, such that the technicians are unaware of group allocation. The statisticians will also be blinded to group labels when analyzing primary outcomes. Through these procedures, any potential assessment or analysis bias is mitigated, preserving the integrity of the comparisons.

### Outcome measures

#### Primary outcome

The primary efficacy outcome is change in glycemic control, as measured by the change in HbA1c from baseline to 12 weeks. HbA1c is a standard indicator of long-term glycemic control, reflecting approximately 3-month average blood sugar levels. A reduction in HbA1c indicates improved diabetes management. The primary comparison will be the mean HbA1c change in the DOE group versus CBE group at the 12-week endpoint.

#### Secondary outcomes

Secondary outcomes were categorized into several relevant domains to comprehensively evaluate intervention impacts:

1. Anthropometric Measures

Body Mass Index (BMI, kg/m²): Evaluates overall obesity status, important in assessing the effectiveness of exercise interventions for weight management.
Waist circumference (cm): Indicates central adiposity and its reduction through structured physical activity, closely associated with cardiovascular and metabolic risk.

2. Cardiovascular Measures

Resting systolic and diastolic blood pressure (mmHg): Monitors cardiovascular risk reduction attributed to regular aerobic and resistance exercises.
Resting heart rate (bpm): A marker of cardiorespiratory fitness, expected to decrease with improved physical conditioning from consistent exercise.

3. Glycemic and Metabolic Measures

Fasting plasma glucose (mmol/L): A direct indicator of short-term glucose metabolism and insulin sensitivity improvements.
Fasting insulin (µIU/mL): Reflects insulin secretion and sensitivity, improvements indicating enhanced metabolic control from regular physical activity.
Homeostasis Model Assessment of Insulin Resistance (HOMA-IR): Calculated measure to evaluate insulin resistance improvements from intervention-induced metabolic adaptations.
Triglycerides (mmol/L): Evaluates lipid metabolism improvements, crucial for reducing cardiovascular disease risk in diabetes.

4. Physical Function Measures

6-minute walk test distance (m): Assesses aerobic endurance and functional capacity enhancement resulting from sustained exercise engagement.
30-second chair-stand test (repetitions): Evaluates lower body muscle strength and endurance improvements through resistance training.

5. Patient-reported Outcomes

SF-36 Physical Component Score (PCS): Reflects the participant’s physical health status improvements linked to regular exercise participation.
SF-36 Mental Component Summary (MCS): Assesses mental health improvements, including stress reduction and psychological wellbeing, potentially enhanced through structured physical activities.

#### Cost-effectiveness parameters

Total intervention-related costs per participant: Captures direct costs (personnel, facility, transportation, and equipment) and app development costs, allowing comparative cost evaluation between digital-based outdoor exercise and clinic-based programs.

Incremental Cost-Effectiveness Ratio (ICER): Determines economic efficiency, comparing incremental costs to clinical effectiveness across key outcomes such as BMI, HbA1c, blood pressure, and physical function metrics.

#### Adverse events and safety outcomes

Throughout the study, we will track any adverse events related to the intervention (such as exercise-related injuries, hypoglycemia episodes during exercise, or cardiovascular events). All serious adverse events will be documented and reviewed by the study’s safety monitoring committee. The number and nature of adverse events in each group will be compared to ensure the digital program does not pose unexpected safety issues relative to standard care.

All outcomes were systematically assessed at baseline, interim (4 weeks), intervention completion (12 weeks), and follow-up (24 weeks) using validated protocols and standardized procedures to ensure data accuracy and comparability.

### Sample size calculation

The sample size is driven by the primary outcome (HbA1c change at 12 weeks) and the non-inferiority design. We assume the clinic-based rehab will yield an HbA1c reduction of approximately 0.4% (absolute % HbA1c) with a standard deviation of 1.0% based on prior exercise trials. We set the non-inferiority margin as 0.4% HbA1c (as justified under primary outcome). Using a one-sided alpha of 0.025 (for non-inferiority) and power of 80%, and assuming equal group sizes, we estimate needing roughly 75 patients per group. To account for up to 25% loss to follow-up by 12 weeks, we plan to enroll 200 patients total (100 per arm).

### Statistical analysis

The primary analysis will follow the intention-to-treat principle, including all participants as randomized, regardless of adherence or dropout. Every effort will be made to obtain follow-up data on all randomized individuals. Participants who withdraw or are lost to follow-up will not be replaced. In the primary analyses, missing outcome data will be handled under the missing-at-random assumption inherent in the mixed-model approach (described below). A complementary per-protocol analysis will be conducted for the primary outcome as a sensitivity analysis, including only those participants who substantially adhered to the intervention protocol​. Non-inferiority for the primary outcome will be concluded if the between-group difference in mean HbA1c change at 12 weeks, along with its 95% confidence interval, lies entirely above –0.4% (the negative of the non-inferiority margin). This corresponds to demonstrating that the digital program is not worse than the clinic program by more than 0.4% HbA1c. If non-inferiority is established, we may also examine whether the digital intervention achieves comparable or even superior outcomes to the clinic intervention as an exploratory measure, although the trial is not explicitly powered for superiority detection.

The primary outcome (HbA1c change) and other continuous outcomes will be analyzed using mixed-effects linear regression models (linear mixed models) to account for repeated measures over time. The model will include fixed effects for treatment group, time (as categorical variable for baseline, 12-week, and 24-week measurements), and the interaction of group * time to assess any differential changes between groups. A random interception for each participant will be included to model within-subject correlation over time. We will adjust baseline values of the outcome (either by including the baseline as a covariate or by appropriately coding time so that baseline is included as the time 0 measurement in the model) to improve estimate precision. The primary comparison of interest is the group difference in outcome change at 12 weeks; this will be obtained from the group*time interaction term at 12 weeks in the mixed model. The estimate of the difference in HbA1c change between groups will be presented with a two-sided 95% confidence interval. For non-inferiority, as noted, the 95% CI will be used to judge against the –0.4% margin. A one-sided test at alpha 0.025 will also be performed for formality of the non-inferiority hypothesis. Secondary continuous outcomes will be analyzed with similar mixed-model frameworks or analysis of covariance (ANCOVA) approaches controlling for baseline values. For categorical outcomes, we will use chi-square tests or logistic regression as appropriate. All hypothesis tests for secondary outcomes will be two-sided with a significant level of 0.05, and confidence intervals will be reported.

Alongside the clinical outcomes, a cost-effectiveness analysis will be performed to compare the economic implications of DOE versus CBE. The analysis will adopt a health system perspective, considering direct costs of the interventions and medical care during the study. We will collect data on the resources used in each arm: for the digital program, this includes costs of the app development (amortized per participant or per use) and personnel time for remote coaching; for the clinic program, costs include facility use, exercise equipment, and staff time for supervising sessions. In addition, healthcare utilization during the 24-week trial will be tracked for each participant to capture medical costs. All costs will be converted to 2025 Chinese yuan (CNY) and, if needed, standardized using purchasing power parity to allow interpretation with common thresholds. The primary measure of cost-effectiveness will be the incremental cost-effectiveness ratio (ICER), calculated as the difference in mean total cost between the digital and control groups divided by the difference in mean effectiveness outcomes between the groups​. To assess uncertainty in the cost-effectiveness results, we will employ non-parametric bootstrap resampling. We will generate bootstrap samples of the participant data and recompute costs, effects, and ICERs to derive 95% confidence intervals for the incremental cost and effect differences.

Statistical significance was determined by two-sided tests with a significance threshold set at p<0.05, performed using SPSS (version 27) and R (version 4.2.0).

## Results

### Participants

**Figure 1:**
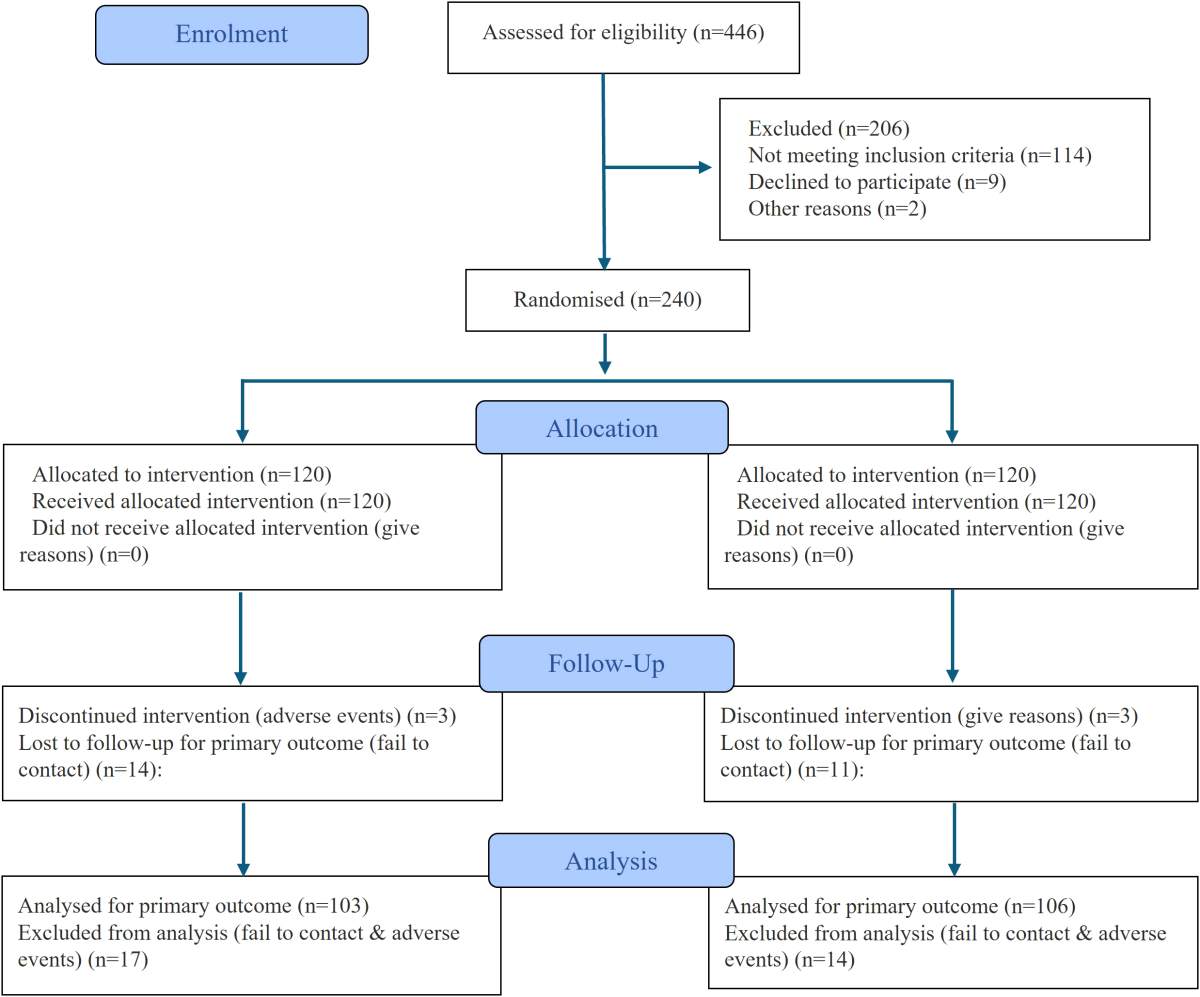
Flowchart of the study.

At baseline, a total of 240 participants were randomized equally into the DOE group and CBE group (n=120 each). Finally, 209 participants were analyzed. The flow diagram of the study was shown in **Figure 1**. The groups were comparable across demographic, medical history, and functional outcome parameters, with no statistically significant differences observed (**Tables 1**). Participants had a mean age of 51 years in the DOE group and 52 years in the CBE group (p=0.156). Male participants comprised 35.00% of the DOE group and 38.33% of the CBE group (p=0.592). Mean BMI was identical at 31 kg/m² in both groups. The majority in both groups had education levels lower than high school and government insurance coverage. Clinical characteristics, including mean diabetes duration and insulin use, were similar. Additionally, baseline HbA1c values were consistent between groups (8 ± 0.52% DOE, 8 ± 0.51% CBE; p=0.303), as were functional measures such as 6-minute walk test distance (DOE 447 ± 81.08 m, CBE 458 ± 72.09 m; p=0.241)​.

**Table 1:** Baseline characteristics.

	Sample characteristic	Digital-based outdoor exercise (N=120)	Clinic-based exercise (N=120)	P value
Baseline characteristics	Male Patients (no. [%])	42 (35.00)	46 (38.33)	0.592
	Age (yr)	51 (4.91)	52 (5.16)	0.156
	BMI (kg/m^2^)	31 (1.45)	31 (1.28)	0.555
	Education level (no.[%])			
	Lower than high school	84 (70.00)	80 (66.67)	0.579
	Equal/higher to high school	36 (30.00)	40 (33.33)	
	Insurance type (no.[%])			
	Government	83 (69.17)	84 (70.00)	0.827
	Commercial	19 (15.83)	21 (17.50)	
	Self-financed	18 (15.00)	15 (12.50)	
	Type of work (no. [%])			
	Labor	68 (56.67)	58 (48.33)	0.196
	Non-labor	52 (43.33)	62 (51.67)	
Medical History	Duration of diabetes (years)	10 (2.75)	11 (2.93)	0.571
	Insulin use (no. [%])	98 (81.67)	103 (85.83)	0.382
	Oral antidiabetic medications (no. [%])	81 (67.50)	84 (70.00)	0.676
	Hypertension (no. [%])	11 (10.68)	12 (11.32)	0.882
	Cardiovascular diseases (no. [%])	9 (8.74)	8 (7.55)	0.753
	Atrial fibrillation (no. [%])	5 (4.85)	5 (4.72)	0.963
	COPD (no. [%])	7 (6.80)	6 (5.66)	0.734
	Arthritis/connective tissue disease (no. [%])	9 (8.74)	11 (10.38)	0.687
	Dyslipidemia (no. [%])	10 (9.71)	12 (11.32)	0.704
	Hypothyroidism (no. [%])	6 (5.83)	7 (6.60)	0.816
Function outcome	HbA1c (%)	8 (0.52)	8 (0.51)	0.303
	Waist circumference (cm)	87 (9.33)	86 (9.50)	0.826
	Resting systolic blood pressure (mmHg)	131 (15.72)	130 (14.40)	0.510
	Resting diastolic blood pressure (mmHg)	89.24 (8.71)	88.86 (9.14)	0.740
	Resting heart rate (bpm)	75 (9.69)	76 (11.59)	0.426
	Fasting plasma glucose (mmol/L)	6 (0.97)	6 (0.89)	0.575
	Fasting insulin (µIU/mL)	7 (1.09)	7 (1.23)	0.523
	HOMA-IR	2 (0.51)	2 (0.52)	0.861
	Triglycerides (mmol/L)	2 (0.30)	2 (0.36)	0.769
	6-minute walk test distance (m)	447 (81.08)	458 (72.09)	0.241
	Chair-stand test (in 30 sec)	15 (2.82)	15 (2.63)	0.493
	SF-36 Physical component score	14 (2.31)	14 (2.17)	0.752
	SF-36 Mental component summary	10 (2.86)	10 (2.72)	0.963

BMI, Body Mass Index; COPD, chronic obstructive pulmonary disease; HbA1c, Glycated Hemoglobin A1c; HOMA-IR, Homeostasis Model Assessment of Insulin Resistance.

### Primary and secondary outcomes

At 12 weeks, the primary outcome, change in HbA1c from baseline, showed no statistically significant difference between the digital outdoor exercise (DOE) and clinic-based exercise (CBE) groups. The effectiveness estimate from the linear mixed-effects model was 0.058% (95% CI: -0.075 to 0.191, p=0.392)​, indicating non-inferiority of the DOE intervention compared to the CBE (**Tables 2**, **3**).

**Table 2:** Changes in outcomes of the DOE and CBE groups at 4,12 and 24 weeks after the surgery (in intention-to-treat population).

Outcome	4 weeks post-surgery			12 weeks post-surgery			24 weeks post-surgery		
	Digital-based outdoor exercise (N=120)	Clinic-based exercise (N=120)	P value	Digital-based outdoor exercise (N=120)	Clinic-based exercise (N=120)	P value	Digital-based outdoor exercise (N=120)	Clinic-based exercise (N=120)	P value
HbA1c (%)	-0.25 (0.06)	-0.25 (0.06)	0.748	-1.04 (0.15)	-0.99 (0.15)	0.012	-1.56 (0.17)	-1.50 (0.17)	0.013
BMI (kg/m2)	-0.26 (0.05)	-0.25 (0.06)	0.121	-0.46 (0.07)	-0.45 (0.09)	0.416	-1.45 (0.21)	-1.45 (0.19)	0.987
Waist circumference (cm)	-2.97 (0.71)	-2.99 (0.77)	0.858	-6.03 (0.88)	-6.01 (0.89)	0.877	-9.06 (1.38)	-8.97 (1.36)	0.620
Resting systolic blood pressure (mmHg)	-5.28 (1.63)	-4.75 (1.44)	0.010	-8.22 (1.60)	-7.96 (1.38)	0.207	-11.14 (1.67)	-10.95 (1.46)	0.363
Resting diastolic blood pressure (mmHg)	-3.04 (0.90)	-2.96 (0.78)	0.478	-4.95 (1.01)	-4.97 (1.01)	0.838	-7.08 (1.11)	-7.02 (1.12)	0.665
Resting heart rate (bpm)	-1.90 (0.91)	-2.35 (4.79)	0.336	-2.81 (1.65)	-3.06 (5.53)	0.645	-3.67 (3.35)	-4.06 (5.49)	0.521
Fasting plasma glucose (mmol/L)	-0.72 (0.39)	-0.74 (0.39)	0.671	-1.03 (0.40)	-1.04 (0.39)	0.769	-1.17 (0.40)	-1.20 (0.40)	0.518
Fasting insulin (µIU/mL)	-1.00 (0.21)	-1.01 (0.19)	0.693	-1.51 (0.25)	-1.49 (0.26)	0.506	-2.01 (0.27)	-1.99 (0.26)	0.704
HOMA-IR	-0.46 (0.12)	-0.47 (0.15)	0.435	-0.66 (0.14)	-0.67 (0.17)	0.417	-0.81 (0.16)	-0.81 (0.20)	0.721
Triglycerides (mmol/L)	-0.11 (0.05)	-0.12 (0.05)	0.579	-0.21 (0.05)	-0.20 (0.06)	0.388	-0.30 (0.06)	-0.29 (0.06)	0.212
6-minute walk test distance (m)	71.80 (38.94)	68.05 (41.75)	0.487	100.44 (39.04)	97.32 (42.08)	0.568	122.60 (37.66)	117.57 (42.36)	0.354
Chair-stand test (in 30 sec)	3.42 (2.54)	3.94 (2.54)	0.123	5.35 (2.62)	6.00 (2.58)	0.062	6.42 (2.55)	7.06 (2.59)	0.064
SF-36 Physical component score	3.94 (1.09)	3.90 (0.96)	0.796	7.01 (1.22)	6.88 (1.11)	0.391	8.96 (1.29)	8.95 (1.18)	0.962
SF-36 Mental component summary	3.96 (0.95)	3.93 (1.13)	0.800	6.95 (1.13)	7.02 (1.12)	0.676	8.96 (1.25)	9.05 (1.26)	0.592

DOE, Digital-based outdoor exercise; CBE, Clinic-based exercise; BMI, Body Mass Index; COPD, chronic obstructive pulmonary disease; HbA1c, Glycated Hemoglobin A1c; HOMA-IR, Homeostasis Model Assessment of Insulin Resistance.

**Table 3:** Effectiveness estimates from linear mixed effects models (in intention-to-treat population).

Outcome	4 weeks post-surgery			12 weeks post-surgery			24 weeks post-surgery		
	Coefficient	95% CI	P value	Coefficient	95% CI	P value	Coefficient	95% CI	P value
HbA1c (%)	0.069	(-0.061, 0.120)	0.294	0.058	(-0.075, 0.191)	0.392	0.050	(-0.083, 0.183)	0.458
BMI (kg/m2)	0.099	(-0.247, 0.444)	0.574	0.100	(-0.244, 0.445)	0.567	0.111	(-0.236, 0.458)	0.530
Waist circumference (cm)	0.280	(-2.112, 2.671)	0.818	0.320	(-2.060, 2.699)	0.791	0.348	(-2.037, 2.733)	0.774
Resting systolic blood pressure (mmHg)	1.008	(-2.809, 4.824)	0.603	1.062	(-2.781, 4.905)	0.587	1.110	(-2.733, 4.953)	0.570
Resting diastolic blood pressure (mmHg)	0.352	(-1.918, 2.621)	0.760	0.392	(-1.891, 2.674)	0.736	0.408	(-1.884, 2.700)	0.726
Resting heart rate (bpm)	-0.899	(-3.549, 1.751)	0.505	-0.877	(-3.468, 1.714)	0.506	-0.845	(-3.352, 1.662)	0.507
Fasting plasma glucose (mmol/L)	0.089	(-0.151, 0.329)	0.465	0.087	(-0.152, 0.326)	0.472	0.095	(-0.142, 0.332)	0.429
Fasting insulin (µIU/mL)	-0.084	(-0.382, 0.214)	0.581	-0.085	(-0.383, 0.212)	0.572	-0.082	(-0.380, 0.216)	0.588
HOMA-IR	0.002	(-0.123, 0.126)	0.979	0.005	(-0.114, 0.124)	0.934	0.006	(-0.108, 0.121)	0.914
Triglycerides (mmol/L)	0.014	(-0.069, 0.097)	0.737	0.012	(-0.071, 0.095)	0.768	0.011	(-0.072, 0.094)	0.797
6-minute walk test distance (m)	-8.798	(-28.722, 11.126)	0.385	-9.195	(-29.531, 11.140)	0.374	-9.022	(-29.672, 11.628)	0.390
Chair-stand test (in 30 sec)	-0.562	(-1.302, 0.177)	0.135	-0.681	(-1.482, 0.121)	0.095	-0.764	(-0.019, 0.018)	0.072
SF-36 Physical component score	0.028	(-0.545, 0.600)	0.924	0.065	(-0.721, 0.851)	0.869	0.019	(-0.632, 0.669)	0.955
SF-36 Mental component summary	-0.003	(-0.721, 0.715)	0.994	-0.058	(-0.803, 0.687)	0.878	-0.102	(-0.869, 0.664)	0.793

BMI, Body Mass Index; COPD, chronic obstructive pulmonary disease; HbA1c, Glycated Hemoglobin A1c; HOMA-IR, Homeostasis Model Assessment of Insulin Resistance; CI, Confidential interval.

For secondary outcomes at 12 weeks, there were no statistically significant between-group differences observed. Body mass index (BMI) changes were similar, with an effectiveness estimate of 0.100 kg/m² (95% CI: -0.244 to 0.445, p=0.567)​. Waist circumference showed minimal difference (0.320 cm; 95% CI: -2.060 to 2.699, p=0.791). Cardiovascular outcomes, including resting systolic blood pressure (1.062 mmHg; 95% CI: -2.781 to 4.905, p=0.587), diastolic blood pressure (0.392 mmHg; 95% CI: -1.891 to 2.674, p=0.736), and resting heart rate (-0.877 bpm; 95% CI: -3.468 to 1.714, p=0.506), also revealed negligible differences​.

Metabolic parameters including fasting plasma glucose (0.087 mmol/L; 95% CI: -0.152 to 0.326, p=0.472), fasting insulin (-0.085 µIU/mL; 95% CI: -0.383 to 0.212, p=0.572), insulin resistance (HOMA-IR: 0.005; 95% CI: -0.114 to 0.124, p=0.934), and triglycerides (0.012 mmol/L; 95% CI: -0.071 to 0.095, p=0.768) were comparable between groups (**Tables 2**, **3**)​.

Physical performance measured by the 6-minute walk test showed an effectiveness estimate of -9.195 m (95% CI: -29.531 to 11.140, p=0.374), and the 30-second chair-stand test indicated a marginal, non-significant difference (-0.681 repetitions; 95% CI: -1.482 to 0.121, p=0.095). Similarly, patient-reported outcomes using the SF-36 indicated minimal and non-significant differences for both the Physical Component Score (0.065; 95% CI: -0.721 to 0.851, p=0.869) and Mental Component Summary (-0.058; 95% CI: -0.803 to 0.687, p=0.878) (**Tables 2**, **3**)​.

Overall, these results demonstrated comparable clinical effectiveness between digital-based outdoor and clinic-based exercise interventions for obese adults with type 2 diabetes mellitus.

### Cost-effectiveness analysis

The average total intervention-related cost per participant was significantly lower in the digital-based outdoor exercise (DOE) group compared to the clinic-based exercise (CBE) group (14,787.30 ± 101.44 CNY *vs*. 17,920.05 ± 3,377.77 CNY; p<0.001) (**Tables 4**, **5**). While the DOE group incurred app development and maintenance expenses (120 CNY per participant), the CBE group had significantly higher costs related to personnel (15,399.31 ± 3,332.13 CNY) and facility usage (392.04 ± 46.77 CNY). Patient transportation costs were also substantially lower in the DOE group (413.03 ± 85.42 CNY) compared to the CBE group (1,989.02 ± 355.46 CNY; p<0.001). The incremental cost-effectiveness ratio (ICER) analysis demonstrated that DOE was cost-saving compared to CBE, with an incremental cost reduction of -3,132.75 CNY per participant. Clinical outcomes showed negligible between-group differences across BMI, waist circumference, systolic and diastolic blood pressures, HbA1c, and other metabolic markers, resulting in cost-effectiveness estimates strongly favoring the DOE intervention. Consequently, digital-based outdoor exercise presented a highly economically favorable alternative to traditional clinic-based exercise, offering comparable health outcomes at significantly lower costs (**Tables 4**, **5**).

**Table 4:** Average total cost per participant during the 24 weeks after the surgery (in intention-to-treat population).

Cost category (CNY)	Digital-based outdoor exercise (N=120)	Clinic-based exercise (N=120)	P value
Intervention-related costs	13960.00 (0)	0.00 (0)	/
Personnel cost (PT time, coaching hours)	0.00 (0)	15399.31 (3332.13)	0.000
App development & maintenance (per patient)	120.00 (0)	0.00 (0)	/
Facility and equipment usage	150.70 (29.27)	392.04 (46.77)	0.000
Resistance bands/Exercise equipment provided	143.56 (67.31)	139.68 (59.94)	0.653
Patient transportation costs	413.03 (85.42)	1989.02 (355.46)	0.000
TOTAL COST	14787.30 (101.44)	17920.05 (3377.77)	0.000

CNY, Chinese Yuan; PT, Physiotherapist.

**Table 5:** Incremental cost-effectiveness ratio (in intention-to-treat population).

	Incremental cost, CNY	HbA1c (%)	BMI (kg/m2)	Waist circumference (cm)	Resting systolic blood pressure (mmHg)	Resting diastolic blood pressure (mmHg)	Resting heart rate (bpm)	Fasting plasma glucose (mmol/L)	Fasting insulin (µIU/mL)
Main analysis - mixed effects	-3132.75	0.050 (-0.083, 0.183)	0.111 (-0.236, 0.458)	0.348 (-2.037, 2.733)	1.11 (-2.733, 4.953)	0.408 (-1.884, 2.700)	-0.845 (-3.352, 1.662)	0.095 (-0.142, 0.332)	-0.082 (-0.380, 0.216)
	HOMA-IR	Triglycerides (mmol/L)	6-minute walk test distance (m)		Chair-stand test (in 30 sec)			SF-36 Physical component score	SF-36 Mental component summary
Main analysis - mixed effects	0.006 (-0.108, 0.121)	0.011 (-0.072, 0.094)	-9.022 (-29.672, 11.628)		-0.764 (-0.019, 0.018)			0.019 (-0.632, 0.669)	-0.102 (-0.869, 0.664)
	BMI (kg/m2)	Waist circumference (cm)	Resting systolic blood pressure (mmHg)	Resting diastolic blood pressure (mmHg)	Resting heart rate (bpm)	HbA1c (%)	Fasting plasma glucose (mmol/L)	Fasting insulin (µIU/mL)	HOMA-IR
Main analysis - mixed effects	-28222.97	-9002.16	-2822.30	-7678.31	3707.40	-62655.00	-32976.32	38204.27	-522125.00
	Triglycerides (mmol/L)	6-minute walk test distance (m)	Chair-stand test (in 30 sec)					SF-36 Physical component score	SF-36 Mental component summary
Main analysis - mixed effects	-284795.45	347.23	4100.46					-164881.58	30713.24

### Adherence and acceptability

Adherence to the prescribed exercise interventions was high and comparable between the digital-based outdoor exercise (DOE) group and the clinic-based exercise (CBE) group (**Supplementary Table S1**). Both groups reported engaging in exercise approximately 3.5 times per week (DOE: 3.5 ± 0.6; CBE: 3.6 ± 0.6, p=0.717). Participant agreement with accepting the allocated exercise plan was similarly high (DOE: 8.4 ± 1.2; CBE: 8.4 ± 1.4, p=0.920). Self-reported compliance with the recommended exercise protocol was consistently high (DOE: 8.3 ± 1.4; CBE: 8.2 ± 1.3, p=0.230). Additionally, participants from both groups indicated substantial agreement that the intervention effectively relieved pain (DOE: 8.6 ± 1.3; CBE: 8.4 ± 1.2, p=0.118), improved function (DOE: 8.5 ± 1.0; CBE: 8.4 ± 1.2, p=0.897), and met overall satisfaction with the exercise protocol (DOE: 9.0 ± 0.9; CBE: 8.8 ± 0.9, p=0.178). These findings suggest that both digital and clinic-based exercise approaches are equally acceptable and well adhered to by obese patients with type 2 diabetes.

### Adverse events

The incidence of adverse events was low and comparable between groups, with 10 (8.3%) participants in the DOE group and 12 (10.0%) in the CBE group experiencing events (**Supplementary Table S2**). Most events were minor and related directly to the exercise intervention, including knee pain, bruising, swelling, minor falls, nausea, dizziness, and anxiety. The most common related event was knee pain (DOE: 5 cases; CBE: 4 cases). Serious adverse events were rare, occurring in only 3 (2.5%) participants from each group. In the DOE group, one serious event (severe muscle sprain) was related to the study therapy. The CBE group reported no therapy-related serious events, although fractures due to falls and severe cartilage degeneration occurred unrelated to the intervention. Participants experiencing serious adverse events were withdrawn from the study. Overall, both exercise modalities demonstrated a favorable safety profile with no significant differences in adverse or serious adverse events between the group.

### Sensitivity analysis

Sensitivity analysis using the per-protocol population showed consistent results with the primary analysis, confirming non-inferiority of the DOE compared to CBE. There were no significant between-group differences across primary and secondary outcomes, while cost-effectiveness results similarly favored the digital-based approach due to lower associated costs (**Supplementary Tables S3**-**S6**).

## Discussion

This randomized trial compared a digitally delivered outdoor exercise program against standard clinic-based exercise in obese adults with T2DM. The results demonstrated that digital intervention was non-inferior to clinic-based exercise in improving key outcomes. Participants in both groups achieved similar reductions in HbA1c and BMI, along with comparable gains in physical fitness and quality of life. Notably, adherence to the digital program was very high, and the intervention proved more cost-effective than the clinic-based approach. These findings suggest that a remotely guided exercise regimen can yield health benefits equivalent to traditional supervised exercise, while enhancing feasibility and efficiency.

Our trial confirms that regular exercise leads to meaningful glycemic improvement in T2DM, and importantly, that a digitally supported program can match the effectiveness of clinic-based exercise. Both groups in our study saw HbA1c declines of similar magnitude, supporting non-inferiority. This aligns with prior studies in China and internationally. For example, Li et al. reported that a fitness app plus wearable system achieved HbA1c reductions indistinguishable from those of supervised center-based exercise ​ (13). Similarly, a U.S. noninferiority trial found an immersive telemedicine platform elicited comparable HbA1c drops to in-person care (14). In our study, both the digital and clinic groups attained clinically significant HbA1c improvements, indicating that remote guidance did not compromise glycemic control. These results reinforce growing evidence that tele-exercise interventions can deliver glycemic outcomes on par with face-to-face exercise programs​ (15).

In terms of weight and metabolic risk, the digital intervention was also non-inferior. Participants in both arms showed significant BMI reductions over the trial. Some earlier studies suggested that unsupervised home exercise might produce less weight loss than supervised sessions​ (16). For instance, an Iranian RCT found clinic-based group exercise led to greater BMI and fat mass reductions than a home-based program, although the home exercise still improved glycemic control and body composition (15). In contrast, our findings indicate that with robust digital support, weight/BMI outcomes can be equivalent to supervised exercise. This is consistent with Li et al’s trial, in which the app-monitored group achieved similar BMI decreases to the control group, while actually attaining a larger reduction in body fat percentage​ (13). The remote monitoring and individualized feedback in our digital program may help patients exercise at the proper intensity, thereby mitigating differences in weight loss seen with less supervised programs. We also observed improvements in blood pressure and lipid profiles in both groups, paralleling the metabolic benefits reported in other exercise interventions​. Notably, comprehensive digital management programs in China (combining exercise, diet, and education) have demonstrated even greater metabolic gains. For example, a 6-month WeChat-based program lowered HbA1c by 1.3% and significantly improved weight and blood pressure versus usual care (15)​. Taken together, the evidence indicates that a well-designed digital exercise intervention can confer broad cardiometabolic improvements comparable to clinic-based approaches.

Physical fitness and functional capacity improved substantially in both groups, with no difference between interventions. We measured significant gains in exercise capacity among digital participants that matched those of the clinic group. This finding mirrors Li et al’s report of greater increases in cardiopulmonary endurance in the app-monitored group compared to controls​ (13). It also echoes the conclusions of a recent scoping review that tele-exercise is as effective as in-person training for enhancing functional capacity and muscle strength in T2DM (5). Furthermore, participants’ self-reported health-related quality of life improved similarly in both groups by the end of the trial. In some prior studies, supervised exercise yielded slightly higher quality-of-life gains than unsupervised programs​, likely due to greater social interaction and support (15). In our trial, however, the digitally supported exercise was able to provide engagement and motivational support sufficient to achieve comparable quality-of-life improvements to the face-to-face program. This encouraging result suggests that interactive features of the mobile platform can capture many of the psychosocial benefits of group exercise. Our findings contribute to the growing consensus that tele-exercise programs can maintain strong impacts on patient-centered outcomes like quality of life, depression, and diabetes distress, rivaling those of clinic interventions​ (14).

Participant adherence to the digital outdoor exercise (DOE) program was notably high, matching that of the clinic-based exercise (CBE) group. Both groups reported similar exercise frequency (3.5 sessions per week), with comparable high levels of satisfaction and willingness to follow recommended exercises. Such strong adherence aligns with previous studies employing digital interventions for chronic disease management, indicating that digital technologies can effectively sustain patient engagement in long-term physical activity programs (13, 14). For example, prior domestic trials using smartphone apps and remote monitoring have achieved adherence rates exceeding 80%, similar to our findings (13). Other studies, including the MOTIVATE-T2D trial, also reported excellent retention (82%) with wearable-supported home exercise programs, reinforcing our results (17).

Several factors contributed to the successful engagement observed in our digital group. The convenience and flexibility of exercising outdoors and at home allowed participants to overcome common logistical barriers, such as transportation and scheduling conflicts. Furthermore, real-time feedback, personalized goal-setting, and regular remote coaching provided by the app appeared to enhance motivation and accountability, elements essential for sustained participation in exercise programs (13, 17). Compared to traditional supervised sessions, the ability to receive immediate digital feedback helped participants maintain consistent exercise intensity and adherence. While prior research suggested reduced adherence in unsupervised home exercise programs, our structured digital intervention with comprehensive monitoring and support effectively mitigated this issue, demonstrating that technology-enabled interventions can maintain engagement comparable to face-to-face supervision (14). These findings suggest that carefully designed digital exercise interventions represent a viable and effective strategy for promoting adherence and sustained engagement among obese adults with type 2 diabetes mellitus (T2DM).

Our analysis demonstrated superior cost-effectiveness of the digital exercise intervention compared to the clinic-based approach. Despite achieving comparable improvements in glycemic control, BMI, physical fitness, and quality of life, the digital program incurred significantly lower total costs per participant. Major cost reductions arose primarily from eliminating personnel supervision, significantly decreasing facility usage expenses, and reducing patient transportation costs. These findings align with previous economic evaluations indicating that digital health interventions can achieve health outcomes similar to traditional methods at reduced costs (18, 19). For instance, an earlier review of physical activity interventions in diabetes highlighted that digitally supported interventions frequently demonstrate cost-effectiveness or even cost savings from a healthcare system perspective (19).

The lower cost observed in our digital approach underscores the economic feasibility of scaling up digital exercise programs, particularly beneficial in resource-limited settings or geographically dispersed populations. By leveraging existing smartphone infrastructure and remote monitoring technologies, digital interventions minimize ongoing expenses related to healthcare professional time and dedicated exercise facilities. This efficiency is particularly important given the increasing economic burden of diabetes management worldwide, especially in healthcare systems facing financial constraints and increasing chronic disease prevalence. Thus, the significant cost advantages of digital-based interventions support their broader adoption and integration into standard care pathways for managing obesity and diabetes. These results suggest that digital exercise interventions represent a cost-effective alternative for health systems seeking sustainable, efficient, and scalable approaches to diabetes care (18, 19).

### Clinical implications

This study’s outcomes have practical implications for diabetes care and public health. First, demonstrating non-inferiority of a digital exercise program means that clinicians can confidently recommend these programs as an alternative to facility-based exercise for obese patients with T2DM. This expands the menu of effective interventions, allowing personalization to patient preferences and circumstances. For patients facing barriers to attending supervised exercise sessions, a structured outdoor program guided by a smartphone could markedly improve access. Our high adherence rates indicate that patients are willing and able to engage with digital health tools for exercise, even in an older, obese population. This is encouraging for broader implementation.

The digital nature of the intervention also means it can reach geographically dispersed or underserved communities. Recent research in China has shown that digital health programs can significantly improve diabetes risk factor control in primary care settings ​ (20). In the SMART Diabetes trial, a multifaceted app-based intervention led to better combined control of HbA1c, blood pressure, and LDL cholesterol than usual care, particularly in rural areas. Our findings complement this by focusing specifically on exercise: a digital exercise regimen can be one key component of holistic digital diabetes management. Health systems could integrate such programs into routine care. The superior cost-effectiveness observed suggests that scaling up digital exercise interventions could free up healthcare resources or allow them to be reallocated to other aspects of diabetes care. Additionally, because our digital program was delivered outdoors, it capitalized on local environments to encourage physical activity, which may have community-wide benefits. There is also potential to combine the digital exercise approach with social support groups or family involvement via the app to further enhance outcomes, which could be investigated in the future. Overall, the study supports a shift toward blended care models in T2DM, wherein patients leverage technology to manage their lifestyle with remote support, reducing the burden on clinic resources without sacrificing efficacy.

### Strengths and limitations

This trial was rigorously designed as a randomized controlled non-inferiority study, directly comparing a novel digital intervention against the current standard of supervised exercise. To our knowledge, it is one of the first RCTs to evaluate a fully digital exercise program in an obese T2DM population, and the sample size provided adequate power to assess non-inferiority across multiple outcomes. Intervention adherence was objectively tracked via the app and wearable sensors, which strengthens confidence in the fidelity of the exercise reporting. We employed validated outcome measures, lending credibility to the findings. Another strength is the pragmatic nature of the intervention, it was tested in real-life conditions with minimal exclusion criteria – which enhances generalizability. Furthermore, conducting a formal cost-effectiveness analysis alongside the clinical trial is a notable strength, as it provides immediate insight into the value of the intervention for decision-makers. High participant retention and adherence in both groups bolster the validity of comparisons, as there was little attrition bias. Finally, the study addresses an important gap in knowledge regarding how digital health solutions can be applied to exercise therapy in diabetes, providing timely evidence in an era of expanding telehealth.

Some limitations should be acknowledged. First, the trial duration was moderate, and longer-term sustainability of the observed benefits is unknown. It remains to be seen whether digital intervention can maintain glycemic control and weight loss over the years, or whether periodic re-engagement or booster sessions might be required. Second, due to the nature of the intervention, participants and providers were not blinded to group assignment. This could introduce bias in patient-reported outcomes. We attempted to mitigate this by blinding outcome assessors for fitness tests and using objective metrics for primary endpoints, but some risk of bias persists. Third, our study population consisted of relatively motivated patients who consented to an exercise program; they may not represent all individuals with T2DM. Thus, there is a potential selection bias, and the results might not generalize to patients with very low health literacy or limited smartphone access. Fourth, while we demonstrated cost-effectiveness, our economic analysis took the healthcare system perspective and did not capture indirect costs or cost savings to patients. Including these might have shown even greater societal benefit of the digital approach. Lastly, we compared the digital program to an active exercise control; there was no group without an exercise intervention. Therefore, while we can conclude non-inferiority to standard exercise, we cannot quantify the full magnitude of exercise benefit versus no exercise from our data (this is well established by prior studies). Despite these limitations, the study provides robust evidence supporting the use of digital exercise interventions in diabetes care.

## Conclusion

In conclusion, a digitally delivered outdoor exercise intervention was non-inferior to clinic-based exercise for improving glycemic control, reducing obesity, enhancing fitness, and boosting quality of life in obese adults with T2DM. The digital approach achieved these outcomes with high adherence and greater cost-effectiveness, highlighting it as a viable and scalable alternative to traditional supervised exercise. These results underscore the potential of mobile health and tele-exercise strategies to expand access to effective lifestyle therapy in T2DM, without compromising efficacy. Wider implementation of such digital programs could help reach more patients, reduce healthcare costs, and ultimately improve long-term diabetes outcomes. Future research should build on these findings by exploring long-term maintenance, integration with comprehensive diabetes management, and applicability to other populations. Our trial adds to the evidence base advocating for technology-enabled interventions as part of chronic disease management in the 21st century, aligning with the CONSORT framework for evaluating digital health solutions in clinical practice.

## Data Availability

The original contributions presented in the study are included in the article/**Supplementary Material**. Further inquiries can be directed to the corresponding authors.
