# Supplementary material for: Digital outdoor exercise program for obese patients with type 2 diabetes mellitus: a non-inferiority randomized controlled trial
Source: Front Endocrinol (Lausanne). 2025 Jul 31;16:1654129. doi: 10.3389/fendo.2025.1654129 (PMC12350124; doi:10.3389/fendo.2025.1654129)
Supplement: Supplementary file 1 [file DataSheet1.docx]

**Supplemental Materials**

**Methods**

**Overview of Interventions**

Participants were randomized to either a Digital Outdoor Exercise Program or a Clinic-Based Rehabilitation Program for a 12-week intervention period. Both programs were matched in overall exercise “dose” (frequency, intensity, and type) consistent with diabetes exercise guidelines. Each intervention combined aerobic and resistance training to achieve approximately 150 minutes per week of moderate-intensity aerobic activity plus regular strength training, as recommended for patients with type 2 diabetes. All participants received an initial orientation session with education on exercise safety, use of equipment (if applicable), and program expectations. They also received written materials about exercise techniques, intensity targets, and precautions. Key components for each group are detailed below.

Digital Outdoor Exercise Program (POE group)

This program delivered a structured exercise regimen remotely via a smartphone app and tele-coaching. Participants were encouraged to perform their workouts outdoors (e.g. walking or jogging in their neighborhood) while being guided and monitored digitally. The intervention was designed to leverage technology to provide professional exercise guidance and supervision comparable to clinic-based rehab, while minimizing barriers like travel and scheduling.

*Weekly Structure and Progression*

The digital program lasted 12 weeks, with exercise frequency of ~5 days per week. The weekly schedule included 3 aerobic-focused sessions (e.g. brisk walking or cycling) and 2 combined sessions that integrated aerobic and resistance exercises. Each session began with a warm-up (~5–10 minutes of light activity and stretching) and ended with a cool-down (~5–10 minutes of stretching). Sessions in the first week were shorter (~20–30 minutes of aerobic activity) and lower intensity to allow safe adaptation. The program progressively increased in duration and intensity approximately every 2 weeks as fitness improved. Below is an outline of the progression and goals over the 12 weeks:

- Weeks 1–2 (Initiation Phase): Emphasis on establishing routine and proper form. Aerobic exercise such as brisk walking is performed ~20–30 minutes per session at a moderate pace (target ~50% of age-predicted maximal heart rate, RPE ~11–12). Light resistance exercises are introduced using body weight or low resistance (e.g. exercises like squats, wall push-ups, or band exercises with minimal tension), 1–2 sets of ~10 repetitions each. The goal is to familiarize participants with the exercises and ensure they can perform them safely and correctly, while achieving roughly 90 minutes of moderate activity per week in this initial phase. Participants are instructed to log activity and note any discomfort or symptoms.
- Weeks 3–4 (Building Phase): Increase aerobic duration to ~30–40 minutes per session by the end of week 4, aiming for ~120–130 total minutes of aerobic activity/week. Intensity is kept at moderate (gradually towards ~60% HR_max or RPE 12–13, “somewhat easy” to “moderate” effort). Resistance training volume increases: e.g. 2 sets of 10–12 repetitions for each exercise, possibly adding light dumbbells or higher resistance band tension if available. New exercises targeting additional muscle groups (e.g. adding lunges or band rows) may be added. The goal for this phase is to improve cardiovascular endurance and introduce more strength work while maintaining good technique.
- Weeks 5–6 (Progressive Phase I): Aerobic sessions progress to ~40 minutes each at a moderate intensity (cumulatively ~150 minutes/week of aerobic exercise by week 6, hitting the minimum guideline target. Intensity may approach 60–70% HRR (heart rate reserve) for some workouts, or RPE around 13 (“somewhat hard”). Participants might incorporate short higher-intensity intervals during walks (e.g. 1–2 minutes of faster walking/jogging interspersed) if capable, to gradually boost fitness. Resistance exercises are further progressed (2–3 sets of 10–15 reps), possibly increasing resistance band tension or adding weight (e.g. using water bottles as weights) for added challenge. By this point, all major muscle groups are being trained (e.g. legs, arms, core) with a variety of exercises. This phase’s goal is to steadily improve exercise capacity and muscle strength as the participants adapt.
- Weeks 7–9 (Progressive Phase II): During weeks 7–8, aerobic exercise duration may extend to ~45 minutes per session, and intensity reaches moderate-to-vigorous levels for brief periods (e.g. incorporating more frequent or longer brisk intervals). By weeks 8–9, some sessions target ~70–75% HR_max (or 60–70% HRR) at peak, corresponding to RPE 13–14 (“moderately hard”). Participants might be jogging or walking briskly uphill if appropriate, to increase intensity. Strength training continues at 2–3 sets per exercise, and resistance can be further increased (heavier band or additional exercises). For example, a participant might progress from wall push-ups to incline push-ups or from body-weight squats to squats with a resistance band. Flexibility and balance exercises can be added after workouts to prevent stiffness and improve functional fitness. The goal in this phase is to challenge participants at a higher intensity and improve their aerobic capacity and muscular endurance, while closely monitoring tolerance.
- Weeks 10–12 (Maintenance/Peak Phase): Final phase where participants sustain the maximum prescribed workload. Aerobic sessions are ~45–60 minutes at moderate intensity, with segments of vigorous effort if tolerated (up to ~80% HRR for short intervals, RPE 15 or “hard” on some days). This might include faster-paced walking, jogging, or cycling intervals, ensuring no adverse symptoms. Resistance training is at its highest volume: up to 3 sets of 15 repetitions of each exercise at the maximum comfortable resistance. By week 12, participants are achieving the full recommended exercise volume (≥150 min/week of aerobic exercise plus 2–3 days of strength training). The aim is to maximize fitness gains while preparing participants to continue regular exercise beyond the trial. Week 12 also serves as a taper/recovery week in some cases, allowing rest before final assessments.

Throughout all phases, the app provides weekly exercise goals and motivational tips. The progression is individualized – if a participant struggles at a given level, the remote coach may adjust the targets to ensure safety. Conversely, if participants adapt quickly, they are encouraged to increase intensity within the prescribed range. This adaptive approach aligns with standard exercise training principles and ADA/ACSM guidelines to ensure gradual overload without injury.

*Exercise Modality and Intensity Details*

Aerobic Training: The primary aerobic modality is brisk walking or hiking outdoors, as it is accessible and safe for obese individuals. Participants are also allowed to choose cycling or jogging if appropriate for their fitness level. Intensity is aimed at moderate level (50–75% of maximal heart rate, or 60–75% HRR) in most sessions, corresponding to a Borg Rating of Perceived Exertion (RPE) of about 12–14. In practical terms, this means the activity is somewhat hard but the participant can still talk in short sentences. As fitness improves, some sessions include short bouts of vigorous intensity (>75% HR_max, RPE 15–16) to further improve cardiovascular fitness, provided participants remain symptom-free. Distance or step count can be used as secondary targets – for example, aiming for ~3,000–5,000 steps during a 30-minute walk in early weeks and increasing to ~7,000–8,000 steps in 45 minutes in later weeks (these targets are adjusted to individual baseline capacity).

Resistance Training: Strength exercises focus on major muscle groups – legs, arms, back, core. The program uses body-weight exercises and resistance bands (exercise bands were provided to participants) since workouts are done at home or outdoors without gym machines. Example exercises include squats, sit-to-stand from a chair, lunges, calf raises for lower body; push-ups against a wall or bench, resistance band rows, biceps curls with bands, and overhead presses for upper body. Participants perform approximately 8–10 exercises per strength session, with an initial intensity that allows ~10–15 repetitions per set with proper form. Intensity is monitored via RPE for resistance exercise as well – aiming for an RPE of ~13 (“somewhat hard”) during the last few reps, which roughly corresponds to a moderate weight (around 50% of one-repetition maximum) for novice individuals. As weeks progress, resistance is increased by adding another set or using stronger bands/weights, so long as the RPE remains in the moderate range (not exceeding 15–16 to avoid undue strain). By the end of the program, participants might be doing 2–3 sets of 15 reps for each exercise with significantly greater resistance than Week 1, reflecting improved strength and endurance. Flexibility exercises (stretching major muscle groups) are included during warm-ups and cool-downs every session to maintain range of motion and reduce injury risk.

*Remote Coaching and Evaluation*

The digital group received substantial remote support to mimic the supervision of a clinic program. The intervention was delivered through a custom mobile application plus scheduled video conferences with an exercise coach. Key aspects of remote guidance and evaluation included:

- Mobile App Guidance: Participants accessed a weekly exercise plan through the app, which listed their recommended workouts, sets/reps, and targets for the week. Instructional videos and written descriptions were provided for all exercises to ensure proper form. The app also featured safety tips (for example, reminders to warm up, stay hydrated, and check blood glucose if on insulin). Participants could refer to these resources anytime (asynchronous support). The app also sent automated reminders before scheduled exercise days and motivational messages afterwards (e.g. congratulating them for completing a session). This approach aimed at keeping participants engaged and adherent between coaching sessions.
- Live Video Orientation: At the start of the program, each participant had a one-on-one video call with a physiotherapist or exercise specialist. This session introduced the exercise regimen, allowed the coach to demonstrate and observe key exercises, and ensured the participant could properly use the app and any equipment (like resistance bands or fitness tracker). It also included testing the technology (video platform) to prevent technical issues later. Baseline vital signs were reviewed (heart rate, blood pressure, blood glucose) and a short functional assessment (such as a few exercise movements or a 6-minute walk in place test) was done to tailor the initial exercise intensity. This orientation ensured participants were comfortable with the digital format and understood how to exercise safely on their own.
- Weekly Tele-Coaching Check-ins: Participants had regular video conferences (teleconference) with their coach to evaluate progress and adjust the program. These occurred once per week (approximately 15–30 minutes each) in the early weeks, and could be group calls (with 3–5 participants per coach) or one-on-one as needed. During these live check-ins, the coach reviewed the participant’s logged activities, asked about any difficulties or symptoms, and provided feedback. Participants could be asked to demonstrate an exercise via webcam to ensure proper technique, or to perform a short fitness task (for example, a partial remote 6-minute walk test or a step-up test) to gauge improvement in endurance. Notably, the 6-minute walk test (6MWT) – a measure of aerobic capacity – can be reliably conducted via telehealth with the participant walking outdoors or in place while supervised through video. In this trial, at the 6-week midpoint, a structured remote evaluation was conducted: participants performed a 6MWT in their home or outdoor setting via video link, and a virtual interview assessed their RPE and any changes in symptoms or medication. This mid-program evaluation allowed the intensity to be recalibrated if necessary. A similar evaluation was done at week 12 (end of intervention) for the digital group in addition to lab-based outcome assessments, to document functional gains in the home environment. The combination of real-time video feedback and data review helped maintain accountability and allowed the exercise prescription to be individualized (for example, increasing walking speed or adding exercises if the participant was not challenged enough, or scaling back if they reported excessive fatigue).
- Communication: Outside of scheduled video check-ins, participants could communicate with the coach or study staff via in-app messaging or phone/email if questions arose. Coaches monitored the app dashboard daily for any alerts (described below) or signs of non-adherence. This ensured that participants in the digital arm had ongoing support despite not meeting in person.

*Monitoring and Adherence Tracking*

Adherence to the digital exercise program was rigorously monitored through the app and wearable technology. Each participant was provided with a wearable activity tracker (or asked to use their own device if compatible) to record physical activity data such as step count, exercise duration, and heart rate. The mobile app automatically logged exercise sessions when synced with the tracker or when the participant manually entered a completed workout. The research team had access to a secure dashboard showing each participant’s activity logs.

- Exercise Logs: Participants were instructed to log every exercise session in the app (with the option of automatic logging via the tracker or manual input). Logged data included duration of activity, perceived intensity (the app prompted the user to input an RPE after each session), and any notes (symptoms or difficulties). Compliance was reviewed weekly. If a participant missed a scheduled session (i.e. no log was recorded on an expected day), the app would send a reminder notification. If multiple sessions were missed or a pattern of non-compliance was detected, the system flagged the coach to follow up. The coach would then call or message the participant to troubleshoot barriers to exercise (e.g. illness, time constraints) and help get them back on track. Maintaining at least 80% adherence (completing ≥80% of prescribed sessions) was set as the goal, which is a common compliance threshold in exercise trials.
- Remote Monitoring Alerts: The digital platform included safety monitoring features. Participants were prompted by the app to report any adverse symptoms experienced during or after exercise (such as chest pain, unusual shortness of breath, dizziness, or injury). They could do this via a quick checklist after logging a session. If any warning symptom was reported, or if the participant indicated feeling unwell, an alert was generated for the study team. Additionally, the app asked users on certain medications to input their blood glucose readings before and after workouts. If a dangerously low blood glucose (hypoglycemia) or very high level was reported, the system would advise the participant to take appropriate action (e.g. consume carbohydrates or postpone exercise) and notify the medical staff. These monitoring steps ensured that any potential issues in the remote setting were quickly identified. Fortunately, in this trial no serious adverse events occurred during digital exercise sessions, but the infrastructure was in place.
- Automated Reminders & Motivation: To promote adherence, the app sent motivational messages and reminders throughout the week. For example, if a participant had not logged any exercise by mid-week, they might receive a friendly message encouraging them to be active, highlighting their goals or past successes. Participants also received brief weekly progress summaries (e.g. “You walked 8 miles total last week!”) to reinforce their achievements. Such behavioral reinforcement, along with the weekly coach check-ins, helped maintain high engagement. In prior studies, personalized messaging and feedback have been shown to improve exercise adherence in diabetes management.

*Safety Protocols for Remote Exercise*

Safety was a top priority in the digital exercise arm, given that participants were exercising outside of a clinical setting. Multiple layers of precautions were implemented:

- Pre-Participation Screening: All individuals underwent medical screening before starting the program to ensure it was safe for them to engage in moderate exercise. A study physician reviewed each patient’s medical history, medication regimen, and recent bloodwork. Participants at higher cardiovascular risk (e.g. history of heart disease) or those with symptoms were given an exercise stress test or referred for cardiology clearance prior to enrollment. Those with uncontrolled hypertension or other contraindications were excluded or treated before proceeding. All enrolled participants met the low-to-moderate risk criteria, meaning they could start a moderate-intensity exercise program without needing continuous ECG monitoring. Each participant’s personal diabetes care provider was informed of their trial participation, and medication adjustments (especially insulin or sulfonylureas) were made if needed to reduce hypoglycemia risk on exercise days.
- Exercise Intensity Monitoring: Participants were equipped with tools to self-monitor during workouts. They were taught how to check their heart rate (using the wrist activity tracker or manually) and given target heart rate zones corresponding to moderate intensity. For example, a 50-year-old might have a target exercise HR of around 110–130 bpm. They were also educated to use the Borg RPE scale (6–20) to gauge effort, aiming for “somewhat hard” (~13) during most of the workout. The combination of HR and RPE allowed participants to regulate intensity. If at any point they felt the exercise was too intense (e.g. struggling to speak, RPE >17) they were instructed to slow down or pause. The app periodically reminded users during aerobic sessions to “check intensity – you should be able to speak a sentence” as a built-in safety cue.
- Home Measurements: To mirror clinic safety checks, digital participants were asked to measure certain parameters on their own. They were provided (or asked to use their own) devices for home monitoring: a blood pressure cuff, a blood glucose meter, and if possible a pulse oximeter. Before each session, participants were told to measure their blood glucose (especially if on insulin/secretagogue medications) and blood pressure. They were given specific cut-off criteria: for example, do not exercise if resting systolic BP >180 mmHg or diastolic >100 mmHg, or if blood glucose <100 mg/dL (5.6 mmol/L) without having a snack. They were educated that if blood glucose was low (<100), they should consume a 15–30 g carbohydrate snack, wait 15 minutes and re-check, ensuring BG rises above 100 mg/dL before starting to exercise. If blood glucose was extremely high (>300 mg/dL) and they felt unwell, they should postpone exercise and contact the study nurse. These guidelines followed ADA recommendations for safe exercise in diabetes. After each session, they were asked to re-check blood glucose to catch delayed hypoglycemia, and to measure blood pressure and heart rate during cool-down. All these self-reported values could be entered into the app. Participants were instructed to contact the study team if any reading was out of range or if they experienced symptoms like dizziness, chest pain, or palpitations during exercise. The remote coaches reviewed the logged vital signs during weekly calls, and any concerning trends (e.g. consistently elevated blood pressure) were flagged for medical follow-up.
- Emergency Protocol: Each participant was informed of an emergency plan. They were advised to always carry a mobile phone during outdoor exercise and, if possible, not to exercise in very remote areas alone. In case of any serious symptom (such as signs of heart attack or severe hypoglycemia confusion), they were to stop exercising immediately and call emergency services. The app had an “Emergency” button that would display their exact GPS location (for those who enabled GPS tracking) and emergency contact info, to assist responders if needed. Fortunately, no such events occurred. Additionally, the study team was available by phone during typical exercise hours if participants had urgent questions. These measures aimed to provide a safety net despite the remote nature of the program.

All participants in the DOE group received training and ongoing support for using the devices and app. At the start of the study, we held an in-person orientation session for the intervention group, where trained research staff provided a hands-on tutorial on operating the wearable heart rate sensor and navigating the exercise smartphone app. During this session, participants practiced using the app (e.g., starting an exercise session, reading their heart rate, and logging activity) with staff supervision to ensure they felt confident. We designed the app to be user-friendly, with a simple interface, large icons/text, and step-by-step prompts, so even participants with limited technical experience could use it. We also provided an illustrated user guide (and quick-start pamphlet) for reference at home.

Despite these measures, we anticipated that some participants might encounter technical issues or have questions. To address this, we set up a technical support system. Participants could reach the study team via a dedicated phone line or messaging chat for any app/device difficulties. Our staff were on-call to help troubleshoot problems such as syncing the heart rate sensor, resetting passwords, or navigating app features. In addition, during the first few weeks, we made weekly check-in calls to participants to ask if they faced any technical difficulties and to proactively assist if needed.

In summary, the digital program provided a comprehensive exercise intervention remotely, pairing flexible outdoor exercise with technology-driven supervision. It achieved a progressive increase in activity tailored to each individual, monitored adherence through digital tools, and maintained safety via education, self-monitoring, and rapid communication channels. This approach sought to overcome traditional barriers (travel, scheduling) while still delivering an exercise dose equivalent to standard care.

Clinic-Based Rehabilitation Program (CBE group)

Participants assigned to the clinic-based rehabilitation arm underwent a standard supervised exercise program at the hospital’s exercise center. This program was designed to mirror the content of the digital intervention (in terms of exercise frequency, intensity, and type) while providing face-to-face supervision and access to facility equipment. The goal was to ensure both groups received an equivalent therapeutic exercise exposure, with the primary difference being the delivery mode (in-person clinic vs. remote outdoor) (Protocol.docx). The clinic program is a typical diabetes and weight management exercise regimen delivered by physiotherapists and exercise specialists.

*Weekly Structure and Progression*

The clinic-based program was 12 weeks long, with a core schedule of 3 supervised exercise sessions per week (on non-consecutive days, e.g. Mondays, Wednesdays, Fridays) at the hospital’s rehabilitation gym. Each supervised session lasted approximately 60 minutes and followed a consistent format (warm-up, main exercise, cool-down), similar to the digital group. To match the digital group’s total weekly activity, clinic participants were also prescribed 2 additional home-based aerobic sessions per week (e.g. walking or cycling on the weekend) (Protocol.docx). They logged these home sessions in a paper diary or mobile app, and they were counted toward the ~5 days/week of exercise. Thus, both groups had a target of ~5 days of activity weekly (3 being directly supervised for the clinic group). The exercise progression in the clinic arm was implemented by the supervising staff, who increased the difficulty roughly every 2–3 weeks as participants’ fitness improved. The progression schedule was flexible based on individual responses, but generally followed this outline:

- Weeks 1–2 (Initiation Phase): Focus on participant orientation and light exercise. Each supervised session begins with ~10 minutes of warm-up (e.g. slow walking or calisthenics). Aerobic training in these weeks might consist of ~20 minutes of continuous exercise at a light-to-moderate intensity (e.g. walking on a treadmill at a comfortable pace or cycling on a stationary bike with low resistance). Resistance training in early sessions emphasizes learning form on the equipment: participants are introduced to weight machines and free-weight exercises targeting major muscle groups. For instance, they might do 1–2 sets of 10–12 repetitions on exercises like leg press, chest press, or seated row with a light weight. Intensity is kept low (approximately 50% of one-repetition max for resistance exercises, and ~50% HRR for aerobics) to accommodate deconditioned individuals. The goal in the first 2 weeks is to familiarize participants with gym equipment, ensure they learn proper exercise techniques under close supervision, and assess their baseline exercise tolerance. Any home aerobic sessions in these weeks are simple walks of 20–30 minutes at an easy pace, just to establish routine.
- Weeks 3–4 (Building Phase): Increase exercise volume and intensity slightly. Supervised sessions now aim for ~25–30 minutes of aerobic exercise at a moderate intensity (e.g. increasing treadmill speed or incline or cycling with moderate resistance to reach ~60% HRR, RPE around 12–13). Resistance training is expanded to 2 sets of each exercise, with weight loads adjusted upward if the participant found the initial weights too easy (targeting an RPE of ~13, or about 60% 1RM). Additional exercises may be added to cover all major muscle groups (for example, adding exercises for smaller muscle groups or core exercises like planks or back extensions as appropriate). By the end of week 4, a typical session might include 30 min of brisk treadmill walking and 20 min of resistance training. The aim is to reach a solid moderate-intensity workout and ensure participants attend regularly. Home sessions on off-days are increased to ~30+ minutes of brisk walking, aiming to accumulate more total minutes of activity.
- Weeks 5–6 (Progressive Phase I): Aerobic duration in supervised sessions is extended toward 30–40 minutes, possibly incorporating variety (e.g. trying an elliptical trainer or a group aerobics class for cross-training one day). Intensity remains moderate; some participants may progress to interval training such as alternating 2 minutes fast/2 minutes slow on the treadmill to safely push their capacity. Resistance training might progress to 3 sets of 8–12 reps for major exercises, and further weight increases as strength improves (maintaining moderate effort, RPE ~14). Flexibility and balance exercises can be introduced during cooldowns to aid those with joint stiffness or balance issues (common in older or obese patients). By week 6, many participants will have achieved the guideline-recommended 150 minutes/week of exercise (combining supervised and home sessions). A mid-program assessment is often scheduled around week 6: clinic staff measure progress in metrics like weight, blood pressure, or a short fitness test (e.g. a 6-minute walk test done in the hallway) to document improvements and motivate participants.
- Weeks 7–9 (Progressive Phase II): These weeks focus on advancing the intensity for greater gains. Aerobic exercise during supervised sessions may incorporate higher intensity intervals or longer continuous exercise. For instance, participants might do 40 minutes on the treadmill including brief jogs or higher incline walking to reach an RPE of 14 (“somewhat hard”) for short periods. Alternatively, if equipment is available, they may try interval circuits (5 minutes each on treadmill, bike, and rowing machine) to keep things engaging. Resistance training could shift to slightly heavier loads aiming for fatigue around 10 repetitions (roughly 70% 1RM for those who can tolerate it), still performed with 2–3 sets. New resistance exercises or more advanced variations (e.g. lunges instead of leg press for those who are able, or adding ankle weights for leg lifts) are introduced to keep stimuli varied. The goal is to maximize improvements in muscle strength and aerobic fitness, under close supervision to maintain safety. Participants are encouraged to continue increasing the pace or distance of their home aerobic sessions as well (e.g. walking faster or for 45–60 minutes on weekends). By this stage, most individuals show measurable improvements in endurance and strength, which the staff use to further encourage adherence.
- Weeks 10–12 (Maintenance/Transition Phase): In the final phase, participants are exercising at a high level of their individualized plan. Aerobic sessions are ~45 minutes at moderate intensity, with some able to sustain higher intensities (e.g. gentle jogging or higher resistance cycling) for extended periods. The focus may shift slightly to preparing participants for transitioning to independent exercise after program completion. This includes teaching them how to self-regulate workouts outside the clinic. For example, in weeks 11–12, the physiologist might simulate a “home workout” scenario during a supervised session – having the participant lead part of their own session (choosing their treadmill settings or guiding themselves through their strength circuit) under observation, to build confidence. Resistance training remains at 2–3 sets but might emphasize proper form and slow, controlled movements at moderately high weight to consolidate strength gains. By week 12, participants ideally reach or exceed 150 minutes/week of aerobic exercise and have increased their strength significantly compared to baseline. The last sessions include a final review of progress and creating a maintenance plan (each participant gets advice on continuing exercise post-trial). Final outcome assessments (at week 12 and later at 24 weeks) are scheduled, where clinic participants will undergo fitness tests and health measurements to gauge the impact of the program.

Throughout the clinic program, progression is personalized: not all participants will reach the same absolute intensity. The supervising staff adjust the speed/incline on cardio machines and the weights on resistance exercises individually, based on heart rate and perceived exertion feedback in each session. If someone experiences difficulty (e.g. excessive fatigue or joint pain), their regimen is modified to lower impact (for instance, switching a participant with knee pain from treadmill to stationary bike). This ensures each person is challenged appropriately without compromising safety.

*Exercise Modality and Intensity Details*

Aerobic Training: The clinic setting allows use of various aerobic exercise machines. The treadmill is commonly used – participants often start with walking and may progress to light jogging if capable. Stationary bicycles (upright or recumbent) provide a low-impact option, especially for those with orthopedic issues. Other equipment like elliptical trainers or rowing machines are introduced for variety and to engage different muscle groups once participants have built some fitness. Aerobic intensity is prescribed and monitored using heart rate and RPE. The target is 60–75% of heart rate reserve (HRR) during aerobic exercise, equivalent to moderate intensity. Heart rate monitors are worn by participants or built into the cardio machines, allowing the physiologist to continuously monitor exertion. For example, if a participant’s target heart rate range is 110–130 bpm, the therapist will adjust treadmill speed or resistance to keep them in that zone. RPE is cross-checked (participants are asked “How hard does it feel on a scale of 6–20?” periodically). In later sessions, short periods of higher intensity (up to ~80% HRR, RPE 15–16) are included for interval training benefits, but always bracketed by recovery periods. The clinic program thus covers a spectrum from moderate to occasional vigorous aerobic exercise, tailored to individual capacity. Duration per session ranges from ~20 minutes initially to 45 minutes or more in later weeks, as tolerated.

Resistance Training: The hospital gym is equipped with weight machines, free weights, and other strength training tools. Under supervision, participants perform exercises for all major muscle groups. Common exercises include: leg press and leg extension (for quadriceps), hamstring curl machine, calf raises (often body-weight or machine), chest press or seated bench press, lat pull-down or seated row for back, shoulder press, and arm exercises (bicep curls, triceps extensions). For core strength, exercises like abdominal crunches or planks are included. Intensity is prescribed at a moderate level (approx 50–70% of 1RM), which is a weight the participant can lift about 10–15 times with effort. In practice, the staff determine a suitable starting weight during week 1 by trial and error – the participant performs an exercise and if they can easily do more than 15 reps, weight is increased; if they struggle to do 8, weight is reduced. The goal is to find a load that yields fatigue around 10–12 reps in the early weeks. That corresponds to an RPE ~13 (“somewhat hard”). Over the course of the program, as participants get stronger, these loads are increased (keeping the rep range similar) to continue challenging the muscles. Sessions typically incorporate 6–10 different resistance exercises, with 1–3 sets each as the program progresses. Between sets, short rests (~1 minute) are given. Proper technique and breathing are emphasized to avoid injury (staff ensure no breath-holding that could spike blood pressure, etc.). Because participants are obese and may have joint issues, any exercise causing pain is substituted with a safer alternative (e.g. reducing range of motion or using elastic bands instead of a heavy weight). By the end of 12 weeks, many participants experience improved strength and are lifting significantly heavier weights or doing more repetitions than at baseline.

Flexibility and Balance: Each supervised session includes stretching major muscle groups during warm-up and cool-down. Gentle static stretches for calves, hamstrings, quadriceps, shoulders, and back are performed (holding ~20 seconds each). Additionally, given some participants may have peripheral neuropathy or balance deficits, therapists incorporate simple balance exercises intermittently – for example, practicing standing on one foot near a support, or heel-to-toe walking along a line. While not a primary focus, these exercises help improve functional mobility and reduce fall risk. The educational talks (described below) also cover foot care, which is important for safe exercise in diabetics (participants are reminded to check their feet for blisters, wear proper shoes, etc., especially when walking a lot).

*Supervision, Monitoring, and Adherence*

By design, the clinic-based program offers close supervision which inherently provides real-time monitoring of exercise performance and safety. Key aspects of how the clinic sessions were run and how adherence was promoted include:

- Group Supervision: Sessions were conducted in small groups of about 4–6 participants per exercise class, led by a physiotherapist or certified exercise physiologist. This group size was chosen to allow each participant to receive individual attention while also benefiting from group camaraderie. The supervisor guides the group through the warm-up, then often splits participants across different machines or stations for the main workout, rotating to assist each person. They ensure everyone maintains proper form in exercises and adheres to their target heart rate/RPE zones. The group setting can boost motivation, as participants often encourage each other.
- Vital Sign Checks: In the clinic, blood pressure and blood glucose were measured before and after exercise for participants where appropriate. At minimum, each supervised session began with a blood pressure check. If a participant had a high reading (e.g. systolic >160 mmHg), they might be asked to sit and relax for a few minutes; if it remained high (>180), the session intensity would be reduced or the participant would be evaluated by the on-site nurse before continuing. For blood glucose, participants on insulin or sulfonylureas were asked to check their glucose with their personal glucometer upon arriving to the gym. If pre-exercise BG was <100 mg/dL, the staff followed the hypoglycemia protocol: provide a 15 g carbohydrate snack and delay exercise until re-check showed BG >100. If BG was >300 mg/dL, exercise was postponed or done at light intensity, and the patient’s physician was consulted if this recurred. These measures mirrored the safety protocols of the digital arm, but with staff directly handling them on site. Heart rate was continuously or periodically monitored during aerobic exercise using chest-strap monitors or the machine’s built-in monitors. Staff also observed for any signs of distress. After exercise, blood pressure was measured again in the cool-down, and any post-exercise glucose if symptoms suggested possible hypoglycemia. Having medical personnel and equipment (oxygen, first aid kit, defibrillator) on hand ensured that any adverse event could be managed immediately. Notably, no serious adverse events occurred in this trial, but several participants did experience mild hypoglycemia episodes which were promptly treated with oral glucose in the clinic, preventing any severe outcomes.
- Attendance Tracking: Attendance for each supervised session was recorded in a registry. If a participant missed a scheduled session, the staff would reach out via phone to check on them and attempt to reschedule a make-up session within the same week. This flexible approach helped maintain the dose of exercise (e.g. if someone missed Monday, they could come Tuesday so they still get 3 sessions that week). Participants were reminded of the importance of consistency. For the home-based sessions (the 2 extra aerobic sessions per week), participants kept a simple exercise diary noting the date, type of activity, and duration (or they could show step count data from a pedometer). These diaries were reviewed each week by the staff, and participants received encouragement or advice based on their entries. If someone was not completing their home walks, staff would problem-solve with them (identifying a safe walking route, scheduling with a family member, etc.). As with the digital arm, an adherence rate of ≥80% of sessions was targeted. The trial achieved high adherence in the clinic group due to the accountability of scheduled appointments and personal contact – most participants attended the majority of their sessions, with missed visits usually due to minor illnesses or travel.
- Progress Review: The rehabilitation team conducted an informal mid-point review (around week 6). They discussed each participant’s progress in a team meeting to decide if any adjustments to the protocol were needed (e.g. if someone was excelling, perhaps increase intensity a bit faster; if struggling, provide extra support). At week 12, participants had a comprehensive assessment (as part of outcome data collection) with blind assessors, but the rehab staff also provided each participant individualized feedback on their improvement (for instance, noting how their treadmill endurance or weight-lifting ability had increased since week 1). This feedback was motivational and reinforced the benefits gained, which is an aspect of monitoring progress.
- Adherence Strategies: The clinic program incorporated several strategies to promote adherence and engagement. Participants became part of a cohort that exercised together, fostering a supportive social environment. They were encouraged to exchange phone numbers or form a chat group to help motivate each other for the unsupervised sessions. Staff frequently praised participants for effort and attendance, creating positive reinforcement. If a participant expressed difficulties (e.g. “I find it hard to come after work”), the team would discuss solutions (like trying a different time slot). In addition, family involvement was encouraged when possible – e.g. a family member could accompany the participant for walks at home, or at least help remind them to exercise. These measures, combined with the structured nature of the program, led to excellent retention.

*Education and Safety in the Clinic Setting*

Safety protocols in the clinic were stringent, given it’s a medical setting, and participants also received education to ensure they could exercise safely both in and out of the clinic:

- Staff and Facility Preparedness: All exercise sessions were supervised by certified professionals trained in basic life support and advanced cardiac life support procedures. An emergency cart (with defibrillator and medications) was available in the rehabilitation gym. Staff rehearsed emergency response drills. Fortunately, no cardiovascular emergencies occurred. Nonetheless, being in a hospital setting meant that in the event of any serious symptom, medical assistance was immediately accessible. This high level of emergency preparedness is a major advantage of clinic-based programs. Minor issues like muscle strains or lightheadedness were handled on the spot (e.g. providing rest and fluids, or applying ice if needed).
- Participant Education (Clinic Setting): The clinic-based program included brief educational sessions as part of the rehabilitation curriculum. Typically, after one of the weekly exercise sessions, a 10–15 minute talk or interactive discussion was conducted by the diabetes educator or physiotherapist . Topics covered each week mirrored those delivered via the app in the digital arm, ensuring both groups received comparable information. Examples of topics are: *“Healthy Eating for Diabetes”*, *“Monitoring Blood Glucose during Exercise”*, *“Foot Care and Injury Prevention”*, *“Stress Management and Motivation”*. Participants had opportunities to ask questions and share experiences. They also received printed handouts summarizing key points (for instance, a handout on hypoglycemia signs and how to treat it, or tips for selecting proper footwear). This educational component reinforced safe exercise practices – for example, the foot care module instructed them to inspect feet daily and wear moisture-wicking socks to prevent blisters, which is crucial for diabetic individuals exercising regularly.
- Hypoglycemia Prevention: Because some participants were on glucose-lowering medications, the staff took proactive steps to prevent hypoglycemia during exercise. In addition to pre-exercise glucose checks and snacks when needed, the timing of exercise sessions was coordinated with meals/medications when possible. Participants were scheduled to exercise at times that minimized conflict with peak insulin action (e.g. avoiding mid-morning sessions for someone who took a high dose of insulin at breakfast, or ensuring they had a mid-morning snack). During exercise, the physiologist stayed alert for signs of hypoglycemia (such as tremors, sweating, confusion). A supply of rapid-acting carbohydrates (glucose tablets and juice boxes) was kept in the gym. Participants were educated to recognize their own symptoms and not to be embarrassed to speak up if they felt “off.” They were also reminded to have a small snack before coming to the session if their pre-exercise BG was on the lower side of normal. As a result of these precautions, hypoglycemic events were rare and mild. The ability to immediately measure blood glucose and administer carbs in the clinic provided a safety net that doesn’t exist in unsupervised settings.
- Injury Prevention: The supervised nature of the clinic program inherently reduced injury risk – exercises were taught with correct form, and staff intervened if a participant was doing something incorrectly. Warm-ups and cool-downs were mandatory. If any musculoskeletal pain arose, the on-site physical therapist would evaluate it and modify exercises accordingly (for example, substituting cycling for a week if someone developed foot pain from treadmill walking). Participants were also advised on listening to their bodies and not pushing through sharp pain. No exercise-related injuries requiring medical care occurred in the study, underscoring the efficacy of these precautions.

**References**

1. Albalawi HFA. The Role of Tele-Exercise for People with Type 2 Diabetes: A Scoping Review. Healthcare (Basel). 2024;12(9):917.
2. Sigal RJ, Kenny GP, Boule NG, et al. Effects of aerobic training, resistance training, or both on glycemic control in type 2 diabetes. Ann Intern Med. 2007;147(6):357-369.
3. Batalik L, Kalanin P, Filakova K, et al. Efficacy of supervised home-based, real-time videoconferencing telerehabilitation in patients with type 2 diabetes: a single-blind randomized controlled trial. J Clin Med. 2022;11(17):4952.
4. Jia W. Diabetes care in China: Innovations and implications. J Diabetes Investig. 2022;13(11):1795–1797.
5. Coyle D, Caffrey HM, O’Neill C, et al. Cost-effectiveness of exercise programs in type 2 diabetes. Int J Technol Assess Health Care. 2012;28(3):228–234.
