# Supplementary material for: Digital outdoor exercise program for obese patients with type 2 diabetes mellitus: a non-inferiority randomized controlled trial
Source: Front Endocrinol (Lausanne). 2025 Jul 31;16:1654129. doi: 10.3389/fendo.2025.1654129 (PMC12350124; doi:10.3389/fendo.2025.1654129)
Supplement: Supplementary file 2 [file Table1.docx]

**Table S1 Patients’ adherence to treatment (in intention-to-treat population)**

| **Outcome measure** | **Digital-based outdoor exercise (N=120)** | **Clinic-based exercise (N=120)** | **P value** |
| --- | --- | --- | --- |
| Number of exercises engaged per week, mean ± SD | 3.5 ± 0.6 | 3.6 ± 0.6 | 0.717 |
| Agreement with the following questions (0 to 10)*, mean (SD) |  |  |  |
| To what extent did you agree to accept the allocated exercise plan? | 8.4 ± 1.2 | 8.4 ± 1.4 | 0.920 |
| To what extent did you do the exercise program as recommended? | 8.3 ± 1.4 | 8.2 ± 1.3 | 0.230 |
| To what extent do you agree that the intervention relieved your pain? | 8.6 ± 1.3 | 8.4 ± 1.2 | 0.118 |
| To what extent do you agree that the intervention improved your function? | 8.5 ± 1.0 | 8.4 ± 1.2 | 0.897 |
| To what extent were you satisfied with the exercise protocol? | 9.0 ± 0.9 | 8.8 ± 0.9 | 0.178 |

N/A, not applicable.

* 0 = strongly disagree, 10 = strongly agree.
