# Supplementary material for: Digital outdoor exercise program for obese patients with type 2 diabetes mellitus: a non-inferiority randomized controlled trial
Source: Front Endocrinol (Lausanne). 2025 Jul 31;16:1654129. doi: 10.3389/fendo.2025.1654129 (PMC12350124; doi:10.3389/fendo.2025.1654129)
Supplement: Supplementary file 3 [file Table2.docx]

**Table S2 Adverse Events and Serious Adverse Events (in intention-to-treat population)**

| **Adverse events** | **Digital-based outdoor exercise (N=120)** | **Clinic-based exercise (N=120)** |
| --- | --- | --- |
| Patients with adverse events (no. [%]) | 10 (8.3) | 12 (10.0) |
| Events unrelated to study therapy (no.) | 4 | 5 |
| Events related to study therapy (no.) | 9 | 12 |
| Type of event (no.) |  |  |
| Involved knee |  |  |
| Pain | 5 (3*) | 4 (2*) |
| Bruising | 3 (2*) | 2* |
| Swelling | 1* | 3 (2*) |
| Other |  |  |
| Fall with minor symptoms | 1* | 3* |
| Nausea and dizziness | 1 | 2 |
| Anxiety | 2* | 3* |
| **Serious adverse events**** |  |  |
| Patients with serious adverse events (no. [%]) | 3 (2.5) | 3 (2.5) |
| Events unrelated to study therapy (no.) | 2 | 3 |
| Events related to study therapy (no.) | 1 | 0 |
| Type of event (no.) |  |  |
| Hip fracture due to fall | 0 | 1 |
| Waist fracture due to fall | 1 | 1 |
| Severe muscle sprain | 1* | 0 |
| Severe cartilage degeneration | 0 | 1 |
| Severe low back pain | 1 | 0 |

*Adverse/Serious adverse events related to study therapy

**Patients with serious adverse events were automatically withdrawn from the study
