# Supplementary material for: Digital outdoor exercise program for obese patients with type 2 diabetes mellitus: a non-inferiority randomized controlled trial
Source: Front Endocrinol (Lausanne). 2025 Jul 31;16:1654129. doi: 10.3389/fendo.2025.1654129 (PMC12350124; doi:10.3389/fendo.2025.1654129)
Supplement: Supplementary file 4 [file Table3.docx]

**Table S3 Changes in outcomes** **of the DOE and CBE groups at 4,12 and 24 weeks after the surgery (in per-protocol population)**

| **Outcome** | **4 weeks post-surgery** | | | **12 weeks post-surgery** | | | **24 weeks post-surgery** | | |  |
| --- | --- | --- | --- | --- | --- | --- | --- | --- | --- | --- |
|  | **Digital-based outdoor exercise (N=103)** | **Clinic-based exercise (N=106)** | **P value** | **Digital-based outdoor exercise (N=103)** | **Clinic-based exercise (N=106)** | **P value** | **Digital-based outdoor exercise (N=103)** | **Clinic-based exercise (N=106)** | **P value** | |
| HbA1c (%) | -0.25 (0.06) | -0.25 (0.06) | 0.704 | -1.03 (0.15) | -0.99 (0.15) | 0.030 | -1.55 (0.17) | -1.50 (0.17) | 0.023 | |
| BMI (kg/m2) | -0.27 (0.06) | -0.25 (0.07) | 0.116 | -0.46 (0.07) | -0.45 (0.09) | 0.419 | -1.45 (0.21) | -1.45 (0.20) | 0.879 | |
| Waist circumference (cm) | -2.99 (0.71) | -2.97 (0.77) | 0.856 | -6.03 (0.88) | -5.98 (0.89) | 0.696 | -9.08 (1.40) | -8.96 (1.37) | 0.547 | |
| Resting systolic blood pressure (mmHg) | -5.22 (1.59) | -4.74 (1.38) | 0.019 | -8.13 (1.57) | -7.98 (1.40) | 0.481 | -11.09 (1.66) | -10.98 (1.45) | 0.623 | |
| Resting diastolic blood pressure (mmHg) | -3.03 (0.90) | -2.94 (0.77) | 0.459 | -5.00 (0.99) | -5.00 (0.95) | 1.000 | -7.07 (1.11) | -7.05 (1.10) | 0.892 | |
| Resting heart rate (bpm) | -1.91 (0.91) | -2.41 (4.94) | 0.320 | -2.79 (1.68) | -3.06 (5.68) | 0.644 | -3.61 (3.41) | -4.17 (5.47) | 0.379 | |
| Fasting plasma glucose (mmol/L) | -0.71 (0.41) | -0.74 (0.39) | 0.663 | -1.02 (0.40) | -1.04 (0.40) | 0.727 | -1.16 (0.41) | -1.20 (0.41) | 0.559 | |
| Fasting insulin (µIU/mL) | -1.00 (0.22) | -1.02 (0.19) | 0.691 | -1.51 (0.24) | -1.48 (0.26) | 0.494 | -2.00 (0.25) | -1.99 (0.27) | 0.751 | |
| HOMA-IR | -0.46 (0.13) | -0.47 (0.15) | 0.344 | -0.65 (0.14) | -0.67 (0.17) | 0.502 | -0.80 (0.16) | -0.81 (0.20) | 0.797 | |
| Triglycerides (mmol/L) | -0.11 (0.05) | -0.12 (0.05) | 0.347 | -0.21 (0.05) | -0.20 (0.06) | 0.526 | -0.30 (0.06) | -0.30 (0.06) | 0.355 | |
| 6-minute walk test distance (m) | 71.50 (36.83) | 67.54 (42.24) | 0.471 | 100.25 (36.94) | 97.66 (42.72) | 0.517 | 121.66 (37.58) | 117.28 (42.81) | 0.433 | |
| Chair-stand test (in 30 sec) | 3.49 (2.56) | 3.94 (2.53) | 0.195 | 5.45 (2.61) | 6.02 (2.63) | 0.116 | 6.41 (2.60) | 7.05 (2.62) | 0.078 | |
| SF-36 Physical component score | 3.94 (1.10) | 3.88 (0.95) | 0.651 | 6.96 (1.22) | 6.81 (1.08) | 0.348 | 8.95 (1.30) | 8.92 (1.19) | 0.876 | |
| SF-36 Mental component summary | 3.95 (0.94) | 3.97 (1.13) | 0.889 | 6.97 (1.12) | 7.04 (1.13) | 0.668 | 9.00 (1.26) | 9.07 (1.26) | 0.705 | |

DOE: Digital-based outdoor exercise; CBE: Clinic-based exercise; BMI: Body Mass Index; COPD: chronic obstructive pulmonary disease; HbA1c: Glycated Hemoglobin A1c; HOMA-IR: Homeostasis Model Assessment of Insulin Resistance
