# Supplementary material for: Digital outdoor exercise program for obese patients with type 2 diabetes mellitus: a non-inferiority randomized controlled trial
Source: Front Endocrinol (Lausanne). 2025 Jul 31;16:1654129. doi: 10.3389/fendo.2025.1654129 (PMC12350124; doi:10.3389/fendo.2025.1654129)
Supplement: Supplementary file 5 [file Table4.docx]

**Table S4 Effectiveness estimates from linear mixed effects models (in per-protocol population)**

| **Outcome** | **4 weeks post-surgery** | | | **12 weeks post-surgery** | | | **24 weeks post-surgery** | | | |
| --- | --- | --- | --- | --- | --- | --- | --- | --- | --- | --- |
|  | **Coefficient** | **95% CI** | **P value** | **Coefficient** | **95% CI** | **P value** | **Coefficient** | **95% CI** | **P value** |  |
| HbA1c (%) | 0.052 | (-0.089, 0.193) | 0.471 | 0.036 | (-0.088, 0.160) | 0.569 | 0.026 | (-0.156, 0.208) | 0.775 |  |
| BMI (kg/m2) | 0.152 | (-0.229, 0.533) | 0.432 | 0.151 | (-0.230, 0.532) | 0.435 | 0.152 | (-0.230, 0.534) | 0.433 |  |
| Waist circumference (cm) | 1.042 | (-1.544, 3.628) | 0.428 | 1.029 | (-1.559, 3.618) | 0.434 | 1.006 | (-1.587, 3.599) | 0.445 |  |
| Resting systolic blood pressure (mmHg) | 1.194 | (-2.864, 0.839) | 0.562 | 1.227 | (-2.841, 5.296) | 0.553 | 1.253 | (-2.821, 5.327) | 0.545 |  |
| Resting diastolic blood pressure (mmHg) | 0.618 | (-1.811, 3.046) | 0.617 | 0.632 | (-1.798, 3.062) | 0.609 | 0.634 | (-1.799, 3.066) | 0.608 |  |
| Resting heart rate (bpm) | -0.965 | (-3.785, 1.856) | 0.501 | -0.957 | (-3.709, 1.796) | 0.494 | -0.881 | (-3.537, 1.776) | 0.514 |  |
| Fasting plasma glucose (mmol/L) | 0.121 | (-0.135, 0.377) | 0.353 | 0.124 | (-0.132, 0.379) | 0.341 | 0.128 | (-0.125, 0.382) | 0.320 |  |
| Fasting insulin (µIU/mL) | -0.075 | (-0.392, 0.243) | 0.643 | -0.085 | (-0.401, 0.232) | 0.599 | -0.086 | (-0.403, 0.230) | 0.591 |  |
| HOMA-IR | 0.014 | (-0.117, 0.146) | 0.829 | 0.016 | (-0.110, 0.142) | 0.800 | 0.015 | (-0.105, 0.135) | 0.804 |  |
| Triglycerides (mmol/L) | 0.020 | (-0.067, 0.107) | 0.653 | 0.017 | (-0.070, 0.104) | 0.696 | 0.015 | (-0.072, 0.102) | 0.730 |  |
| 6-minute walk test distance (m) | -6.393 | (-27.971, 15.185) | 0.560 | -5.857 | (-27.853, 16.139) | 0.600 | -5.392 | (-27.678, 16.893) | 0.634 |  |
| Chair-stand test (in 30 sec) | -0.507 | (-1.255, 0.241) | 0.183 | -0.622 | (-1.457, 0.214) | 0.144 | -0.696 | (-1.452, 0.061) | 0.071 |  |
| SF-36 Physical component score | -0.202 | (-0.821, 0.418) | 0.522 | -0.162 | (-0.795, 0.470) | 0.613 | -0.173 | (-1.141, 0.795) | 0.705 |  |
| SF-36 Mental component summary | -0.086 | (-0.823, 0.652) | 0.819 | -0.105 | (-1.016, 0.807) | 0.821 | -0.114 | (-1.073, 0.845) | 0.814 |  |

BMI: Body Mass Index; COPD: chronic obstructive pulmonary disease; HbA1c: Glycated Hemoglobin A1c; HOMA-IR: Homeostasis Model Assessment of Insulin Resistance; CI: Confidential interval.
