# Supplementary material for: Digital outdoor exercise program for obese patients with type 2 diabetes mellitus: a non-inferiority randomized controlled trial
Source: Front Endocrinol (Lausanne). 2025 Jul 31;16:1654129. doi: 10.3389/fendo.2025.1654129 (PMC12350124; doi:10.3389/fendo.2025.1654129)
Supplement: Supplementary file 6 [file Table5.docx]

**Table S5 Average total cost per participant during the 24 weeks after the surgery (in per-protocol population)**

| **Cost category (CNY)** | **Digital-based outdoor exercise (N=120)** | **Clinic-based exercise (N=120)** | **P value** |
| --- | --- | --- | --- |
| Intervention-related costs | 13960.00 (0) | 0.00 (0) | / |
| Personnel cost (PT time, coaching hours) | 0.00 (0) | 15375.47 (3305.50) | 0.000 |
| App development & maintenance (per patient) | 120.00 (0) | 0.00 (0) | / |
| Facility and equipment usage | 150.13 (28.41) | 390.63 (46.48) | 0.000 |
| Resistance bands/Exercise equipment provided | 142.72 (67.65) | 139.34 (60.51) | 0.704 |
| Patient transportation costs | 410.49 (86.23) | 1985.19 (359.84) | 0.000 |
| TOTAL COST | 14783.33 (100.96) | 17890.63 (3344.54) | 0.000 |

CNY=Chinese Yuan: PT=Physiotherapist.
