# Supplementary material for: Digital outdoor exercise program for obese patients with type 2 diabetes mellitus: a non-inferiority randomized controlled trial
Source: Front Endocrinol (Lausanne). 2025 Jul 31;16:1654129. doi: 10.3389/fendo.2025.1654129 (PMC12350124; doi:10.3389/fendo.2025.1654129)
Supplement: Supplementary file 7 [file Table6.docx]

**Table S6 Incremental cost-effectiveness ratio (in per-protocol population)**

|  | **Incremental cost, CNY** | **HbA1c (%)** | **BMI (kg/m2)** | **Waist circumference (cm)** | **Resting systolic blood pressure (mmHg)** | **Resting diastolic blood pressure (mmHg)** | **Resting heart rate (bpm)** | **Fasting plasma glucose (mmol/L)** | **Fasting insulin (µIU/mL)** |
| --- | --- | --- | --- | --- | --- | --- | --- | --- | --- |
| **Main analysis - mixed effects** | -3132.75 | 0.026(-0.156, 0.208) | 0.152(-0.230, 0.534) | 1.006(-1.587, 3.599) | 1.253(-2.821, 5.327) | 0.634(-1.799, 3.066) | -0.881(-3.537, 1.776) | 0.128(-0.125, 0.382) | -0.086(-0.403, 0.230) |

**Table 5 continues**

|  | **HOMA-IR** | **Triglycerides (mmol/L)** | **6-minute walk test distance (m)** | **Chair-stand test (in 30 sec)** | **SF-36 Physical component score** | **SF-36 Mental component summary** |
| --- | --- | --- | --- | --- | --- | --- |
| Main analysis - mixed effects | 0.015(-0.105, 0.135) | 0.015(-0.072, 0.102) | -5.392(-27.678, 16.893) | -0.696(-1.452, 0.061) | -0.173(-1.141, 0.795) | -0.114(-1.073, 0.845) |

**Table 5 continues**

|  | **BMI (kg/m2)** | **Waist circumference (cm)** | **Resting systolic blood pressure (mmHg)** | **Resting diastolic blood pressure (mmHg)** | **Resting heart rate (bpm)** | **HbA1c (%)** | **Fasting plasma glucose (mmol/L)** | **Fasting insulin (µIU/mL)** | **HOMA-IR** |
| --- | --- | --- | --- | --- | --- | --- | --- | --- | --- |
| **Main analysis - mixed effects** | -20442.76 | -3088.77 | -2479.89 | -4901.10 | 3527.01 | -119511.54 | -24275.78 | 36131.40 | -207153.33 |

**Table 5 continues**

|  | **Triglycerides (mmol/L)** | **6-minute walk test distance (m)** | **Chair-stand test (in 30 sec)** | **SF-36 Physical component score** | **SF-36 Mental component summary** |
| --- | --- | --- | --- | --- | --- |
| **Main analysis - mixed effects** | -207153.33 | 576.28 | 4464.51 | 17961.27 | 27257.02 |
